# Normalized risk-based evaluation of machine learning–based classification models: A multiclass approach with applications in medical devices

**DOI:** 10.1177/03000605261486667

**Published:** 2026-09-28

**Authors:** Julius Wiggerthale, Martin Haimerl, Christoph Reich

**Affiliations:** 1 Faculty I, Computer Science & Applications, 120263 Furtwangen University, Germany; 2 Faculty II, Engineering & Technology, 120263 Furtwangen University, Germany

**Keywords:** Machine learning, multiclass classification, performance metrics, risk-based assessment, risk management, regulatory requirements

## Abstract

**Objective:**

Machine learning is increasingly integrated into safety-critical domains such as medical applications. In this context, regulatory frameworks require the assessment and minimization of risks associated with incorrect model predictions. However, classical evaluation methods often focus on quantifying error frequencies, which do not reflect the heterogeneous impact of different types of errors. To address this deficiency, we elaborate an approach for assessing machine learning–based multiclass classification models that conforms to regulatory requirements in the field of medical devices. Additionally, we aim to demonstrate its applicability in a concrete application scenario as an exemplary reference.

**Methods:**

Herein, we provide weighted balanced accuracy $WBA_{n}$ as a risk-based approach for assessing machine learning models for multiclass classification. $WBA_{n}$ is systematically based on regulatory requirements for medical devices. It incorporates core properties such as the representation of risks as a multiplicative combination of likelihood and severity and the relationship between development and real-world scenarios. We demonstrate the application of $WBA_{n}$ using X-ray based detection of lung diseases as a reference scenario.

**Results:**

Our analysis shows that $WBA_{n}$ achieves a risk-based assessment in the lung disease scenario as a reference. $WBA_{n}$ systematically operationalizes the definition of risk from corresponding regulatory requirements and allows the assessment to be adapted according to risk levels that have to be elaborated in the context of the risk-management process.

**Conclusion:**

Although this study uses only a single use case to demonstrate the practical applicability of our approach, it shows the impact of a risk-based approach when assessing the performance of machine learning multiclass classification models in medical applications. Without such an approach, regulatory requirements cannot be implemented in a fully comprehensive way. Consequently, the clinical impact cannot be adequately assessed when applying the model in real-world scenarios.

## Introduction

Beyond its utilization in consumer-related products, machine learning (ML) is increasingly being integrated into industrial and scientific applications. For example, ML-based inspection systems often surpass traditional systems in terms of accuracy and robustness.^1,2^ Thus, they provide valuable opportunities in fields that are more sensitive to errors and safety issues. Medical applications are also increasingly being addressed by ML-based techniques. ML models are already able to achieve better performance than medical experts in specific tasks, such as the diagnosis of skin cancer or retinal diseases.^3–6^ In particular, this applies to classification tasks, which are the core topic of this study.

ML is also being utilized in certain medical devices, that is, concrete products addressing medical tasks. However, their usage still remains limited, especially in the European Union (EU).^7^ This is partially due to the stringent regulatory requirements that must be met. For instance, the Medical Device Regulation (MDR)^8^ and the EU artificial intelligence (AI) Act^9^ must be adhered to before ML-based medical devices can be placed on the EU market. Beyond many other aspects, the MDR and AI Act requirements include a thorough assessment of the performance of ML models as well as an appropriate implementation of risk-management (RM) processes as core elements. In the context of medical devices, RM is an obligation that has to be performed across the entire lifecycle of the product.^8,10,11^ Thus, the consideration of risks has to be an integral component of ML model development, including data acquisition, training, validation/tuning, (In this study, we use the term “validation” in the terminology usually applied in the ML context. This refers to the tuning of model hyperparamters to optimize the model. We include the word “tuning” as a second term to make this relationship more accessible. Please note that the term “validation” is used differently in the context of product development and medical devices. Here, it refers to confirmation, through the provision of objective evidence, that the requirements for a specific intended use or application have been fulfilled,” as defined in ISO 9000:2015.) and final assessment steps as well as the rating of outcomes during the operational phase. This requires the integration of risk-based considerations into the evaluation metrics used for the assessment of ML models.^11^

Usually, harmonized standards are utilized to implement the corresponding regulatory requirements. For RM, International Organization for Standardization (ISO) 14971^10^ is the main reference, accompanied by ISO/Technical Report (TR) 24971^12^ and British Standards Institution (BSI)/Association for the Advancement of Medical Instrumentation (AAMI) 34971^13^ as additional references for its implementation. However, there is currently no clear guidance that systematically integrates the requirements for the assessment of ML models with the basic aspects of RM. This deficiency was elaborated on with a focus on binary classification tasks.^11^ However, the basic regulatory requirements for RM have to be considered.

According to these RM requirements, risk is defined as the combination of the likelihood/probability of harm and its severity.^8,10^ Although this definition is central to the development of medical devices, many existing ML evaluation practices rely exclusively on error-based metrics that do not incorporate the heterogeneous impact of different error types.^11^ Metrics such as (balanced) accuracy, sensitivity/specificity, positive predictive value (PPV), F1-score, and receiver operating characteristic (ROC) curves/area under the ROC curve reflect misclassification frequencies but fail to express their associated clinical consequences.^14^ A comprehensive list of standard assessment methods for classification tasks is already available.^15,16^ In many medical use cases, however, the risk associated with a false negative (FN) (e.g. missed diagnosis of a severe disease) differs fundamentally from that of a false positive (FP). As a result, classical evaluation metrics are often insufficient for risk-based model assessment in safety-critical domains.

This requires consideration of the impact of different error types, for example, by appropriate weighting. Weighted evaluation metrics that include such an approach already exist. Some of them are tailored to specific types of ML models.^17–19^ Other approaches are more general.^14,20–22^ Additionally, some of them focus on binary classification, whereas others are formulated more generally and include multiclass classification tasks.

Basically, these weighted metrics make use of distinct misclassification costs for each class, which are usually defined in a cost matrix. Sometimes, a utility-based approach is used, in which a utility is assigned to each potential outcome. In this regard, utility can be considered a kind of negative cost—and vice versa.

Often, these weighted metrics are applied to deal with highly unbalanced datasets.^14,20,21^ In principle, they can also be used to address unbalanced consequences associated with different error types.^14^ However, these approaches are not consistently built on a risk-based framework, as required according to RM principles for medical devices as provided in ISO 14971^10^ and ISO/TR 24971.^12^ They do not systematically apply the definition of risk as a combination of the probability and severity of potential harms.

Weighted metrics are closely related to the area of cost-sensitive learning (CSL), as CSL refers to the application of weighted objectives/loss functions to the development of ML models on a cost-sensitive basis. This can be applied at different stages of development. Basically, there are two main approaches to CSL: direct approaches and meta-learning approaches.^14^ Direct approaches work at the algorithm level. Here, the target of model training shifts from minimizing total error to minimizing costs.^20^

Various techniques have been proposed in this category. Examples include modified decision trees^17^ or k-nearest neighbor (KNN) algorithms^19^ that are directly tailored to specific types of models as well as targeted, cost-sensitive loss functions, such as weighted cross-entropy loss,^23,24^ which can be applied more generally.

In contrast to direct approaches, meta-learning approaches either adapt the data (preprocessing) or the model output (postprocessing). For example, instance weighting is a preprocessing method. Here, samples from different classes are assigned distinct weights.^21^ A common method in postprocessing is thresholding. Thresholding adapts a decision threshold to minimize costs^20,21^ in a specific setting, for example, according to a specific risk profile for a medical decision.

CSL requires appropriate adaptations of weights and thresholds to be derived based on the corresponding data and use context of ML-based medical applications. This can be challenging because it requires quantifying the impact of decisions and types of errors.^11,14^ For this purpose, ISO 14971 and ISO/TR 24971 allow a semi-quantitative approach that partitions levels of likelihood and severity of harm into discrete categories. From a technical perspective, one issue associated with CSL—especially direct approaches—is the danger of overfitting when using inappropriate cost matrices.^14^

Concrete examples of weighted objectives/metrics that can be used are average cost ( AC ),^18^ weighted kappa score $\kappa_{\omega }$ ,^25^ or cost-weighted accuracy ( CWA ).^26^ For AC , the costs of all errors are aggregated using a cost matrix, where AC is the sum of all expected costs. In AC , the costs are not normalized. This makes interpretation and comparison of the outcomes of different models difficult. Additionally, it is strongly affected by the proportion of positive and negative samples in a dataset, as it does not consistently account for class imbalances in a risk-based way. The weighted kappa score $\kappa_{\omega }$ measures the alignment between a model’s predictions and the ground truth while taking distance costs into account. These distance costs are related to weight factors defined for the case in which an instance *i* of class *i* is classified as class *j*. $\kappa_{\omega }$ is also very sensitive to class distributions.^25^ Beyond that, it directly scales with the magnitude of weights, which makes comparison difficult. In general, the $\kappa_{\omega }$ are difficult to interpret and relate to the actual risks of errors.

CWA is defined as a weighted sum of all potential outcomes and their associated utilities. Potential outcomes again refer to the probabilities of cases in which an instance *i* of class *i* is classified as class *j*. Even though CWA is based on utilities instead of costs, this can easily be translated. The authors who introduced CWA normalize the values relative to an inactive classifier that always predicts the default class.^26^ This normalization strongly depends on the range of basic CWA outcomes. This leads to highly individual scores for each particular use case and makes comparison between use cases difficult. Also, CWA is affected by class probabilities because it uses absolute numbers of samples rather than relative metrics such as FPR and FNR . Beyond that, the score measures weighted accuracy and does not provide a consistent translation of the definition of risk as a combination of the likelihood and severity of potential harms.

A recently proposed metric, weighted balanced accuracy ( WBA ), addresses several of these issues by linking model performance directly to the expected risk (ER) for binary classification tasks.^11^ WBA provides normalized, comparable scores that reflect both the probability and severity of misclassifications and enable risk-optimal threshold selection. However, WBA in its current form is limited to the binary case^11^ and does not apply to multiclass settings. However, a multiclass approach is necessary when not only the occurrence of a disease is important but also the identification of its different types.

To address these limitations, the main objectives of this study are to extend the WBA metric to a multiclass setting and demonstrate its applicability and impact in practical applications. In other words, we elaborated the $WBA_{n}$ metric as a normalized metric designed to operationalize the structure required by ISO 14971.^10^ Within our approach, we make use of a multidimensional cost matrix that represents the different class constellations and consequently follows regulatory requirements regarding RM for medical devices. Based on these goals, we define $WBA_{n}$ as a risk-based metric that retains the desirable properties of the binary formulation while becoming applicable to arbitrary numbers of classes. This includes an explicit relation to ER, systematic integration of class distributions/imbalance, and normalization to values between 0 and 1 to allow direct comparison between different ML models.

To exemplarily illustrate the practical applicability, we apply the approach to the training and assessment of ML models for a concrete use case addressing a multiclass classification task. For this purpose, the detection of lung diseases in X-ray images was utilized. An open-access dataset from the Kaggle database was used. In principle, this represents a retrospective observational/cross-sectional study. However, the study itself is not the main objective of the study. Instead, it aims to demonstrate the application of the risk-based methodology to this particular exemplary scenario. This includes a comparison of the results based on the $WBA_{n}$ metric with those of existing weighted metrics. Additionally, the impact of changes in the weights in the cost matrix is demonstrated. Furthermore, conformance with regulatory requirements for risk management in the field of medical devices is assessed.

Our analysis focuses only on the evaluation of ML models. It does not address the inclusion of risk-based metrics in model training, such as the aforementioned CSL approaches. Although this should be considered an extension toward a fully comprehensive integration of risk-based approaches into the development of ML-based medical devices, this was beyond the scope of this study.

## Materials and methods

The applied methods follow three steps. First, we provide the necessary background regarding evaluation metrics for classification tasks for the definition of risk-based metrics. Second, we introduce the $WBA_{n}$ metric for the risk-based assessment of ML models addressing multiclass classification tasks. Third, we apply the $WBA_{n}$ approach to a concrete medical scenario and describe the methods used to analyze the results of this application.

### Background regarding risk-based assessment of classification models

#### Basic types of classification models

In ML, there are two basic types of classification models. For binary classification tasks, a scalar-output model can be used. Here, the model predicts one score, and this score can be compared with a threshold *s* to determine the class of an instance. In contrast, vector-output models are usually applied when addressing multiclass applications. These models predict one score for each class. The predicted class is the one that obtains the highest score.^22^ This basic distinction is important because it demonstrates that postprocessing options such as thresholding are limited to specific conditions, in particular, binary cases. It is not applicable in multiclass settings. This has to be considered regarding the development of appropriate ML models that need to follow regulatory requirements for RM and the development of medical devices. This is also important when developing methods for the assessment of ML models in multiclass settings, as an adjustment cannot be performed using thresholding. Especially in this case, the direct integration of risk-based approaches into the assessment of ML models is an important step.

#### Outcomes in binary classification

For binary classification (e.g. the detection of a potential disease), one class is usually denoted the negative class, and one class is denoted the positive class. A sample of the positive class is denoted a true positive ( TP ) when classified correctly and a FN otherwise. For a sample of the negative class, the appropriate notations are true negative ( TN ) and (*FP*). These four possible situations form the basis for metrics in binary classification tasks.

Standard metrics such as accuracy, PPV , F1 scores, and AUROC are fundamentally based on these central case scenarios.^15,16^ They are also core elements of weighted metrics and risk-based assessment methods, in which the outcome scenarios are combined with the corresponding costs reflecting the impact of the particular cases.^11,14^ Basically, the terms TP , FP , TN , and FN denote the frequencies of the cases. If normalized by the number of cases, these values are transformed into probabilities as follows.

- *False-positive rate (* FPR *).* Share of samples of the negative class classified as the positive class, given as follows:
$$FPR=\frac{FP}{FP+TN}$$
- *False-negative rate (* FNR *).* Share of samples of the positive class classified as the negative class, given as follows:
$$FNR=\frac{FN}{FN+TP}$$

Basically, these values represent class-conditional probabilities. More specifically, FPR=P(FP|N) is the probability that a wrong prediction/a positive result is predicted in a case in which the actual result is negative.

#### Class imbalances and its relationship to real-world scenarios

The described values depend on the given circumstances and the phase in which the evaluation is applied. During model development, the values usually refer to the amount or percentage of outcomes relative to the amount of data in the dataset used for training, validation/tuning, or testing of the model. Thus, they do not directly relate to the expected outcomes in the real-world setting, as the distribution between the classes often intentionally deviates between model development and the real-world scenario. This is due to the importance of balanced datasets during development.

In most applications, positive and negative cases should be represented equally to avoid biases (e.g. a lack of robustness for one type of outcome) when training the models. Conversely, the underlying distribution often has a clear tendency toward one class in the actual application scenario. For example, positive samples are often underrepresented in concrete medical use cases such as screening tasks, where the probability of a disease, that is, the prevalence, is rather low, often substantially below 1.0%.^11^

Consequently, the performance observed on a (balanced) training dataset, measured by FP or FN , cannot directly be translated to real-world scenarios. These values need to be adapted according to the relationship between the distributions of the datasets used during model development and the real-world scenario. Subsequently, these relationships are denoted by the following:

-
$R_{P}=\frac{P}{P_{real}}$ for the positive cases
-
$R_{N}=\frac{N}{N_{real}}$ for the negative cases

where $P_{real}$ and $N_{real}$ are the numbers of (expected) positive and negative cases, respectively, in the real-world scenario. Notably, the terms “positive” and “negative” refer to the actual situation and not to the prediction of an ML model.

#### Regulatory requirements for risk-based assessment

According to the regulatory requirements, medical devices need to be developed in a way that minimizes risks or reduces them to an acceptable level.^8,10,12^ This has to be achieved through appropriate RM activities throughout the entire lifecycle of the device, including the development steps. For ML-based medical devices, this means that the assessment of the corresponding models needs to consider the risks associated with the use of the model. Based on this, the assessment needs to be based on the estimated risk, including the different types of errors and their impact.^11^

For medical devices, risk is defined as the combination of likelihood and severity.^8,10,12^ More precisely, ISO 14971^12^ proposes the definition of risk as a multiplicative combination, that is, risk is equal to likelihood multiplied by severity.

Here, likelihood is the probability that a particular harm occurs, and severity refers to a grade reflecting the impact of the harm when it occurs. For inclusion in a metric, these terms need to be quantified, that is, we need concrete numbers for the particular levels of likelihood and severity.

Alternatively, this may be provided in a semi-quantitative way, according to ISO/TR 24971.^12^ This means that likelihoods and severities may be categorized into discrete levels. Often, a factor of 10 is used for this purpose, that is, the difference between two levels of likelihood is a factor of 10 . For example, one level may represent a probability of 1.0% and the next level is 0.1% . Based on this, the next level of severity also often represents a step by a factor of 10. This is due to the aforementioned multiplicative combination, as well as the usual property that the antidiagonals in the risk matrix according to ISO/TR 24971^12^ are considered to represent constant risk levels.^11^ According to ISO 14971^10^ and ISO/TR 24971,^12^ the estimation of risks, including probabilities and severities, may be performed by an expert panel with sufficient experience regarding the particular application. In particular, this can be applied when more comprehensive clinical data are missing.

As a side note, BSI/AAMI 34971^13^ provides further information about ML-specific risks in the context of medical devices. However, it does not change the basic approach to establishing risk levels. Thus, it has a limited impact on this study, which focuses on a basic concept for performing risk-based assessment of ML-based classification models. Additionally, the EU AI Act^9^ basically applies. However, the RM requirements are similar to the approach in the MDR.^8^ Additionally, sectoral regulations take the lead regarding the implementation of certain requirements, such as RM, as defined in the AI Act. Thus, these additional legislations and guidelines have a minor impact on the fundamentals of a risk-based framework for the assessment of ML-based classification models.

#### Influence of the real-world scenario

As already mentioned, these basic ingredients for the definition of risks need to be related to the actual medical application, that is, the real-world scenario. In particular, this needs to consider the application context in which the ML model is applied.^8,10^ This means that the severities of the potential harms have to be rated according to the potential impact of the device in this scenario. Harm basically refers to a negative clinical impact on the patient (or another involved person). For diagnostic tasks, this may, for example, be related to the progression of a disease (e.g. in the case of a FN) or additional interventions imposed on the patient (e.g. in the case of a FP).

Additionally, the likelihood has to be rated according to the concrete application context. For example, the likelihood may differ between general screening for a disease and a diagnostic test that is applied based on a specific suspicion. Usually, the likelihood of detecting a disease is substantially higher in a dedicated diagnostic test, for example, when there is a specific suspicion, compared with general screening. In the latter, the prevalence (that is, the overall probability) of the disease in the addressed population is a central factor. In general, the likelihood of a potential harm is a multiplicative combination of the probability that an included patient has the disease under examination and the probability that the test delivers a false result, that is, harm is equal to probability multiplied by severity. For example, in the binary case, the overall likelihood $l_{FN}$ of a FN is the prevalence “*Prev”* of the disease in the diagnosed group multiplied by the probability that the test produces a FN result.

$$l_{FN}=Prev\;\cdot \;FNR\;=\frac{P_{real}}{P_{real}+N_{real}}\cdot FNR$$

#### ER—basic terminology

This leads us to the following definition of ER. As a first step, we elaborate this for the binary case. Later, this approach is extended to the multiclass situation. To simplify this step, we already start by using general class numbers 0,1,⋅⋅⋅,n−1 for an arbitrary but fixed number *n* of distinct classes. In the binary case, this corresponds to the classes 0 , representing a negative case, and 1 , representing a positive case. Additionally, we need to distinguish between the actual situation and the predicted outcome, both of which can take these values. Based on this, we obtain pairs (i,j) , where *i* represents the class according to the actual situation and j represents the class corresponding to the outcome predicted by the model. In the binary case, this means that the following:

-
(0,1) represents a false-positive case ( FP );
-
(1,0) represents a (*FN*) case;
- The cases (i,j) with i=j represent correct predictions (*TP*, *TN*).;
- For *i* ≠ *j*, we also use the term “confusion,” as it describes an incorrect assignment between these two classes. This coincides with the notion of the confusion matrix, which represents the number of cases in which an actual class i is assigned to class *j*.

Based on these definitions, we elaborate on the basic components for defining ERs. Basically, we need the numbers and rates of the particular class constellations as well as variables for representing the impact of the particular scenarios.

We start by providing variables representing the impact of the particular outcomes. For this purpose, we define a cost matrix $C=(c_{i,j}{)}_{i,j=0}^{n-1}$ representing the impact of the particular cases, that is, $c_{i,j}$ quantifies the cost for cases in which a sample of actual class *i* is predicted as class *j* by the model. For example, $c_{1,0}$ represents the cost for the (*FN*) case. Following the WBA approach,^11^ we focus on the error cases, as these situations basically carry the major load regarding the potential risks. Thus, we assign zero impact when the prediction is correct, that is, $c_{i,j}\;=0$ in cases where *i = j*.

Regarding the numbers and rates, we use the following terminology. The number of cases in which the model assigns an actual class *i* to the predicted class *j* is denoted by $a_{i,j}$ . The overall number of cases in a given class according to the actual situation is denoted by the following:

$$a_{i}=\overset{n-1}{\underset{\;j=0}{\sum }}\;a_{i,j}$$

Based on this, the conditional probabilities that class j is predicted for an actual class i can be calculated as follows:

(1) $$p_{i,j}=\frac{a_{i,j}}{a_{i}}$$

For example, FNR is calculated as follows:

$p_{0,1}=\frac{a_{0,1}}{a_{0}}=\frac{a_{0,1}}{a_{0,0}\;+\;a_{0,1}}$ .

#### ER—transfer to the real-world scenario

These definitions refer to the situation in the particular development step for the model, for example, training, validation/tuning, or testing. This means that the numbers are calculated according to the given datasets for this step and the corresponding distribution of the data. This includes the class distribution *a_i_*, as well as the numbers of outcomes *a_i,j_*. Notably, the $p_{i,j}$ values depend on the model and not directly on the class distribution in the dataset.

For the actual likelihood $l_{i,j}$ , these numbers need to be transferred to the situation of the real-world application of the model. For this purpose, we define the distribution of classes in the real-world scenario as $d_{i}$ , that is, $d_{i}$ represents the probability that a random candidate in a given scenario or reference dataset belongs to the actual class *i*. Based on this, the likelihood $l_{i,j}$ can be calculated, representing a situation in which the model predicts class *j* for a patient of the actual class *i* when it is used in the designated context. This results in Equation 2:

(2) $$l_{i,j}=d_{i}\cdot p_{i,j}=d_{i}\;\cdot \;\frac{a_{i,j}}{a_{i}}$$

#### ER—definition

These probabilities/likelihoods now have to be combined with the cost matrix. First, we clarify that we use the variables $c_{ij}$ for the costs when considering the class distribution of the dataset used in the development of the model (training, validation/tuning, or testing). Based on this definition, ER can be calculated as the sum of the products of likelihoods and costs across all cases. This is given in Equation 3.

(3) $$ER=\overset{1}{\underset{i=0}{\sum }}\;\overset{1}{\underset{\;j=0}{\sum }}\;l_{ij}\cdot c_{ij}=\overset{1}{\underset{i=0}{\sum }}\;\overset{1}{\underset{\;j=0}{\sum }}\;p_{ij}\cdot d_{i}\cdot c_{ij}\;$$

Because we focus on the $p_{ij}$ values (e.g. FPR and FNR ) during development, we integrate the class distributions *d_i _*of the real-world scenario into a modified cost matrix $\hat{C}=({\hat{c}}_{i,j}{)}_{i,j=0}^{n-1}$ with the following:

$${\hat{c}}_{i,j}=d_{i}\;\cdot \;c_{ij}$$

Based on this definition, the ER can be expressed as follows:

(4) $$ER\;=\overset{1}{\underset{i=0}{\sum }}\;\overset{1}{\underset{\;j=0}{\sum }}\;p_{ij}\cdot {\hat{c}}_{i,j}\;$$

#### Minimization of ER

This ER needs to be minimized to achieve a risk-optimal solution. The minimization should be performed in relation to the relevant parameters. For example, the optimization can be performed with respect to the optimal threshold parameter when the risk-based approach is utilized in a binary classification scenario that includes a threshold-based postprocessing approach. In this case, ER *,* $c_{ij}$ , and ${\hat{c}}_{i,j}$ also depend on such a threshold *s*.^11^ In other cases, the optimization has to be performed, for example, with respect to the model parameters during training or the hyperparameters during model validation/tuning.

The minimization of ER is invariant when using a monotone transformation of the final risk values. For example, this allows linear transformations of the form $\tilde{ER}\;=a\;\cdot ER\;+b$ . For a≠0 , the minimization turns into a maximization.^11^

The authors of the study presenting WBA ^11^ use this property to reduce the definition of ER in the binary case to a single parameter $c_{\;fn}$ that represents the risk ratio between false-positive and (*FN*) cases. This defines the weighted balanced accuracy (*WBA*) metric as follows:

(5) $$WBA=1-\frac{FPR\;+c_{\;fn}\cdot FN}{1\;+c_{\;fn}}$$

The defined WBA metric ranges from 0 to 1 , where 0 indicates the worst possible classification model with maximum ER, and 1 indicates the perfect classification model with 0 ER.

In general, a linear transformation can be utilized to normalize the ER to a range between 0 and 1 . Based on this, it can be decided whether to minimize costs or risks or, alternatively, maximize an accuracy or utility value as a kind of inverse cost value. The latter can be achieved when the transformation parameter *a* is below 0 . As already mentioned, both approaches are equivalent with regard to the comparison of optimization parameters when a fixed transformation is used.

### Introduction of ${WBA}_{n}$ as a risk-based metric for multiclass classification

#### Requirements and basic properties of WBA_n_

Based on these observations, we derive the following properties that should be achieved when defining $WBA_{n}$ as a risk-based metric for multiclass classification models:

1. The $WBA_{n}$ metric shall result in an assessment value that reflects *ER* for the given scenario.
2. ER shall refer to the definition of risk as a combination of the likelihood and severity of harm, according to the regulatory references.^10,12^ Additionally, ER shall be defined using the multiplicative product between probability and severity in terms of a quantitative or semi-quantitative approach.
3. The $WBA_{n}$ metric shall be applicable to multiclass scenarios with an arbitrary number of classes. It should systematically extend the WBA metric in this direction, that is, the WBA metric should represent a special case of the $WBA_{n}$ metric for binary scenarios.
4. The $WBA_{n}$ metric shall reflect the real-world scenario, including a systematic integration of class distributions in the real-world setting and the development environment.
5. The $WBA_{n}$ metric should be normalized to a range between 0 and 1 to enable a direct comparison between different ML models.

#### Definition of $WBA_{n}$

In multiclass settings, it is not possible to define a single risk ratio that is fully equivalent to $c_{\;fn}$ as defined in the previous study^11^ for the binary case. Instead, we focus on the normalization property of WBA when extending it to the multiclass scenario. For WBA, division by $1\;+c_{\;fn}$ in Equation 5 ensures that the score is properly normalized to the range [0,1]. Additionally, $1\;+c_{\;fn}$ represents the maximum $ER_{max}$ that can be achieved by the classification model. Using this observation, WBA can be rewritten in the form

$$WBA=\frac{ER_{max}\;-\;ER}{ER_{max}}$$

According to the equivalence of ER with regard to linear transformations, we use an equivalent version of ER as defined in Equation 4. This leads to the following:

(6) $$WBA=\frac{ER_{max}\;-\;ER}{ER_{max}}=\frac{ER_{max}\;-\;\sum_{i=0}^{1}\;\sum_{\;j=0}^{1}\;p_{ij}\cdot {\hat{c}}_{i,j}\;}{ER_{max}}$$

Please note that we directly use the modified cost matrix ${\hat{c}}_{i,j}$ to include the relationship to the real-world scenario. This was not explicitly used in the previous study^11^ and thus represents a further extension implemented in $WBA_{n}$ . Based on this, the definition in Equation 6 can be directly converted to a multiclass setting with *n* classes:

(7) $$WBA_{n}=\frac{ER_{max}\;-\;ER}{ER_{max}}=\frac{ER_{max}\;-\;\sum_{i=0}^{n-1}\;\sum_{\;j=0}^{n-1}\;p_{ij}\cdot {\hat{c}}_{i,j}\;}{ER_{max}}$$

To actually calculate $WBA_{n}$ , the calculation of the maximum $ER_{max}$ for multiclass classification is required. For this purpose, we introduce the notation $j_{*}(i)={\mathrm{argmax}}_{\;j=0}^{n-1}\;c_{ij}$ as the class achieving the maximum misclassification cost for an actual class *i* across all predicted classes *j*. Based on this, the maximum ER is obtained by a classifier that predicts the class $j_{*}(i)$ for each actual class *i*. This leads to the following:

$$ER_{max}\;=\overset{n-1}{\underset{i=0}{\sum }}\;d_{i}\cdot c_{ij_{*}(i)}$$

Equivalently, the modified cost matrix $\hat{C}$ can be used to calculate $ER_{max}$ :

(8) $$ER_{max}\;=\overset{n-1}{\underset{i=0}{\sum }}\;{\hat{c}}_{ij_{*}(i)}$$

This calculation can then be substituted into Equation 7, finalizing the definition of $WBA_{n}$ as a risk-based metric for the multiclass setting that fulfills the following characteristics:

- The metric $WBA_{n}$ is based on ER.
- Risk is defined as a multiplicative combination of the likelihood and severity of harm, that is, probabilities $l_{ij}$ and costs $c_{ij}$ , or equivalently, $p_{ij}$ and costs ${\hat{c}}_{ij}$ .
- The metric extends the $WBA_{n}$ metric to the multiclass setting for an arbitrary number of classes.
- The metric includes the relationship to the real-world scenario based on the class distributions $d_{ij}$ .
- The metric is normalized to a range between 0 and 1.

### Experimental Setup to Demonstrate the Application of ${WBA}_{n}$

#### Basic characteristics of the study design

To demonstrate how $WBA_{n}$ applies to real-world applications, we conducted experiments using a specific medical application scenario. This study did not involve any new acquisition of data from human participants. It relied exclusively on a publicly available, fully anonymized dataset that does not permit re-identification of individuals. Such secondary use of anonymized, publicly available data do not constitute research on identifiable human subjects. Consequently, ethics committee approval and informed consent were not required. Additionally, the previously published datasets were collected according to the Declaration of Helsinki, under Institutional Review Board (IRB) approval and/or explicit exemption from IRB review.^27–30^ Based on this, our analysis was conducted in accordance with the principles of the Declaration of Helsinki and the rules regarding the secondary use of anonymized data therein.

We utilized the chest X-ray dataset for the diagnosis of lung diseases that is available on Kaggle (https://www.kaggle.com/datasets/muhammadrehan00/chest-xray-dataset).^31^

Formally, this represents a retrospective observational/cross-sectional study. No specific hypothesis was addressed regarding the concrete results for the metrics used. Instead, this example served to illustrate the practical applicability of the $WBA_{n}$ metric and compare it with other weighted evaluation metrics. This demonstrates the steps that have to be performed to set up the cost matrix and apply the $WBA_{n}$ metric to a concrete use case. This addresses the main objective of the study, that is, the definition of the $WBA_{n}$ metric as a normalized metric that is designed to operationalize the structure required by ISO 14971.^10^

Regarding the study design and data preparation, we did not perform any additional steps. We basically used the existing dataset as provided. Thus, there was no specific reporting of missing data and no specific measures to deal with missing data. Within the particular datasets, there were no missing data because only the X-ray images were used. No specific measures for loss to follow-up were applied. No specific subgroup analysis was performed. No dedicated analysis of the cases at the different steps of case selection was provided that could be provided in a flow diagram. Thus, no particular reasons for nonparticipation at each stage had to be reported. In general, the reporting of this study conforms to the Strengthening the Reporting of Observational Studies in Epidemiology (STROBE) guidelines.^32^

To demonstrate the impact of a risk-based assessment based on the $WBA_{n}$ , the analysis in this study includes a comparison with existing metrics. Additionally, the dependency of the $WBA_{n}$ metric on specific parameters of the (modified) cost matrix was analyzed. Furthermore, a sensitivity analysis was performed to demonstrate the robustness of the approach. Based on this, the analysis provided represents a first proof of concept regarding the $WBA_{n}$ approach and does not provide a comprehensive analysis of the outcomes for the particular medical scenario used as a reference.

#### Dataset characteristics

The dataset used consisted of X-ray images belonging to the classes normal (N), pneumonia (P), and tuberculosis (T). It was divided into predefined training, validation/tuning, and test datasets. An overview of the class distribution per split is provided in Table 1.

**Table 1.** Class distribution in the dataset subsets.

Dataset	Normal	Pneumonia	Tuberculosis
Train	7261	4674	8513
Validation	900	570	1064
Test	925	580	1064

Data were recorded across different countries and institutions. The dataset was created from the following different smaller datasets:

- The Dataset of Tuberculosis Chest X-rays Images;^28^
- The Labeled Optical Coherence Tomography (OCT) and Chest X-ray Images for Classification;^27^ and
- The dataset used in a study on reliable tuberculosis classification.^29^

The most important institutions and programs that recorded and collected the images were the National Library of Medicine (NLM; USA), the Radiological Society of North America (RSNA), the National Institute of Allergy and Infectious Diseases (NIAID; Belarus), and the NIAID TB portal program.^33^ No specific eligibility criteria were applied in this setting.

The sources used to create the dataset differ markedly in origin and acquisition conditions. Therefore, the class labels are not evenly distributed across these sources. The pneumonia class was derived exclusively from Labeled Optical Coherence Tomography (OCT) and Chest X-Ray Images for Classification,^27^ consisting of pediatric radiographs in the anterior–posterior (AP) projection from a single center in China, with diagnoses graded by two to three expert physicians. The tuberculosis class was derived from the other datasets, comprising predominantly adult radiographs in the posterior–anterior (PA) projection. The dataset in the study on reliable tuberculosis classification^29^ is an aggregate of the NLM Montgomery and Shenzhen collections, the NIAID TB dataset, the RSNA CXR dataset, and the Belarus dataset, representing different countries, including the USA, China, and Belarus. The data from the Dataset of Tuberculosis Chest X-rays Images^28^ were collected at a single hospital in Pakistan. The normal class was drawn from all three sources. Table 2 summarizes the resulting characteristics. No detailed demographic information was available for the data.

**Table 2.** Characteristics of the source datasets aggregated in the composite dataset.

Source	Contributed classes	Patient population	Projection	Acquisition origin
27	Normal, Pneumonia	Pediatric (1–5 years)	Anterior–posterior	Single center, China
29	Normal, Tuberculosis	Adult	Posterior–anterior	USA, China, Belarus (multiple sources)
28	Normal, Tuberculosis	Not reported	Not reported	Single hospital, Pakistan

The aggregation of different datasets introduces heterogeneity across acquisition site and country, imaging equipment, native resolution and file format, projection, and patient age. For the assessment of the models, it has to be considered that class membership was confounded with patient population and acquisition protocol: pneumonia cases are exclusively pediatric AP radiographs, whereas tuberculosis cases are predominantly adult PA radiographs. As pediatric and adult thoracic anatomy differ substantially, a model may, in part, separate the classes by population- or protocol-specific features rather than the underlying pathology. Beyond that, the class counts of the dataset available on Kaggle exceed the reported sizes of the cited sources, indicating that data augmentation and/or sampling from the larger underlying data pools was applied during its creation. As the per-image provenance is not documented for the aggregated dataset, the images cannot be considered fully statistically independent. This has to be considered when interpreting the bootstrap confidence intervals (CIs) reported in this study.

#### Training and evaluation of ML models

The dataset served as a reference dataset for demonstrating the application of the $WBA_{n}$ approach in a concrete medical application. To examine the application of $WBA_{n}$ , we trained different models (VGG16,^34^ ResNet18,^35^ and ResNeXt50^36^) and then compared the resulting models regarding $WBA_{n}$ as well as average cost ( AC ),^18^ weighted kappa score $\kappa_{\omega }$ ,^25^ and cost-weighted accuracy ( CWA )^26^ as existing weighted metrics. Additionally, we calculated the resulting (unweighted) accuracy levels. The computation of any weighted metric was based on the modified cost matrix $\hat{C}$ that was derived using the $WBA_{n}$ approach. This was used as a compromise because CWA , $\kappa_{\omega }$ , and AC are not actually designed to operationalize the structure required by ISO 14971.^10^ They do not actually refer to concrete levels of risks but represent general costs without a clear relationship to risks.

As already mentioned, we used a VGG16 model,^34^ a ResNet18 model,^35^ and a ResNeXt50 model^36^ for the comparison. All models were initialized with ImageNet weights. The hyperparameters used for training the particular models are documented in Table 3.

**Table 3.** Hyperparameter used to train the models in the multiclass application scenario.

Parameter	VGG16	ResNet18	ResNeXt50
Batch size	16	32	32
Optimizer	Adam	Adam	Adam
Learning rate	0.001	0.001	0.001
Training epochs	50	50	50
Early stopping after	5	5	5

To demonstrate the impact of $WBA_{n}$ , we applied the described methodology to the following two test scenarios:

- Test Scenario #1 using a cost matrix derived from a concrete clinical setting; and
- Test Scenario #2 demonstrating the dependency of model selection on systematically varying particular parameters, that is, weights in the modified cost matrix.

This means that the cost matrices and, subsequently, the assessment according to the $WBA_{n}$ metric were derived for these concrete settings. For this purpose, we used the following order of the classes:

- Class 0: N (normal);
- Class 1: P (pneumonia); and
- Class 2: T (tuberculosis).

For each scenario, the normalization constant for $WBA_{n}$ had to be calculated according to the maximum $ER_{max}$ . In Scenario #1, we additionally performed two types of sensitivity analysis: (a) the cost structure was perturbed by random noise and (b) ranking stability across bootstrap samples was calculated to examine how robustly the proposed metric ranks different models in slightly different subsets of the test dataset. The details of the two scenarios are described below.

#### Test scenarios including risk estimation and additional testing

In this section, we describe the test scenarios, including the estimated risks in these concrete situations. The estimation was performed based on information from literature, following a semi-quantitative approach. The relevant standards, ISO 14971^10^ and ISO/TR 24971,^12^ allow such estimations to be performed based on a consensus of experts when reliable quantitative data are not available. The following analysis mimics this approach. Although this study did not actually include a panel of experts for this estimation, data from the literature served as a proxy for this purpose.

#### Test Scenario #1

The first scenario was based on the definition of a clinically motivated cost matrix ${\hat{C}}_{\left[1\right]}$ . The scenario refers to a clinical situation in which persons with a suspected lung disease are sent to an X-ray-based test for pneumonia. In these cases, tuberculosis may be identified as a secondary finding. We assumed that the test is applied in developed countries in an emergency setting where patients suspected of having a nontraumatic pulmonary infection undergo an X-ray examination.

For this situation, we estimated the prevalences from the literature:

- The prevalence of pneumonia, that is, *d*_1_, was estimated to be approximately 10% ,^37^ as this reference directly reflected the described scenario.
- The prevalence of tuberculosis, that is, *d*_2_, was estimated as 1% .^38^ Here, we did not find direct numbers for the given real-world scenario. Instead, we used a surrogate in which patients were hospitalized with community-acquired pneumonia. In this setting, a tuberculosis rate of approximately 1% was reported.^38^ We argue that these two scenarios basically have similar characteristics, that is, patients with lung problems without a direct suspicion of tuberculosis.
- The prevalence of normal cases, that is, $d_{0}$ , can then be set to the remaining share of approximately 90% .

This defined the basics for the likelihood/probability of the risks. The values $d_{i}$ had to be integrated into the modified cost matrix to finally achieve a proper relationship to the class-conditional probabilities $p_{i,j}$ .

As a next step, we defined the severity levels. In this case, we applied a semi-quantitative approach according to ISO/TR 24971^12^ using five severity levels. For each level, we had to assign a cost factor that achieves the proper combination with the likelihood values. As discussed, we used a factor of 10 between the levels.^11^ This approximately matches the relationships between the probability levels $d_{0}$ vs $d_{1}$ vs $d_{2}$ in this scenario, which decrease by approximately of factor of 10 between the classes. Thus, one step up by one level in probability is compensated by one step down by one level in severity. Concretely, we used the five levels of severity provided in Table 4.

**Table 4.** Severity levels and associated cost factors using a semi-quantitative approach according to ISO/TR 24971.^12^

Severity level	Description	Cost factor
Critical	Results in development and/or progression of a critical/potentially life-threatening injury or disease	1000
Major	Results in development and/or progression of a substantial injury or disease requiring medical or surgical intervention	100
Moderate	Results in development and/or progression of a moderate injury or disease requiring medical or surgical intervention	10
Minor	Results in minor injury or disease (potentially including mental health issues) without the need for further medical or surgical intervention	1
Negligible	Results in inconvenience or minor temporary discomfort	0.1

ISO: International Organization for Standardization.

Based on these severity levels, we had to rate all outcome constellations (i,j) . This was assessed in relation to the potential clinical consequences as follows:

- Case (1,0) , that is, P classified as N

Untreated pneumonia can eventually lead to serious complications, especially in high-risk groups. Usually, the risk remains manageable when the test is used in an emergency setting where disease progression is monitored appropriately. Additionally, other persons may become infected when the disease is not detected. Thus, this case was rated as major, that is, the cost factor was set to $c_{1,0}=100$ .

- Case (0,1) , that is, N classified as P

Patients may receive unnecessary antibiotics for a limited period. This may have potential side effects but usually does not require additional treatment to compensate for these effects. This case was rated as minor, that is, $c_{0,1}=1$ , as the side effects usually do not go beyond the level of a minor injury or disease.

- Case (2,0) , that is, T classified as N

This may lead to unrecognized progression of a critical disease and potentially irreversible lung damage. Additionally, other persons may become infected when the disease is not detected. Based on this, we rated this case as critical, that is, $c_{2,0}=1000$ .

- Case (0,2) , that is, N classified as T

This may result in unnecessary isolation/stigmatization of the patient and eventually also the initiation of therapy or additional potentially invasive examinations. However, this usually should be detected. Based on this, it is assumed that only temporary effects occur without the need for major additional treatment. Thus, this case was rated as moderate, that is, $c_{0,2}=10$ , as the patient is impacted, at least with regard to psychological effects.

- Case (1,2), that is, P classified as T

Here, the main risk is that pneumonia medication is applied too late. Based on this, effects similar to those in case (1,0) are likely to occur. Additionally, inappropriate measures/treatment for tuberculosis may be initiated. Thus, this case was rated as major, that is, $c_{1,2}=100$ .

- Case (2,1), that is, T classified as P

Patients may receive inadequate or delayed specific therapy, with a continued risk of transmission. Again, unrecognized progression of the disease may occur. However, in this case, the patient receives treatment and is followed up. Thus, the situation was not rated as critical but as major, that is, $c_{2,1}=100$ .

- All remaining cases (i,j) with i=j, that is, correct predictions

In these cases, no negative effect was assumed, and thus, $c_{i,j}=0$ . This completed the definition of the use case. Based on these ratings, the risk matrix was set to the following:

$$C_{\left[1\right]}=\left(\begin{array}{ccc} 0 & 1 & 10\\ 100 & 0 & 100\\ 1000 & 100 & 0 \end{array}\right)$$

Combined with the class distributions/prevalences *d_i_* for this concrete setting, the modified cost matrix was set to the following:

$${\hat{C}}_{\left[1\right]}=({\hat{c}}_{i,j}{)}_{i,j=0}^{n-1}=(d_{i}\cdot c_{i,j}{)}_{i,j=0}^{n-1}=\left(\begin{array}{ccc} 0 & 0.9 & 9\\ 10 & 0 & 10\\ 10 & 1 & 0 \end{array}\right)$$

We applied additional methods to assess the statistical robustness of the results. To demonstrate that the proposed metric is robust to changes in the training dataset, we performed k-fold cross-validation, in which we trained k = 10 models on different folds of the dataset. We computed $WBA_{n}$ and other weighted metrics for each fold. Mean values and 95% CIs were calculated as outcomes for all metrics.

Additionally, we drew B=1000 bootstrap samples of size N=2500 with replacement from the test set and calculated the probability that a given architecture was ranked as the best model. This served to assess ranking stability across different subsets of the test population. To also examine robustness to changes in the cost structure, we calculated the likelihood that a given model was ranked as best model by the different weighted metrics when scaling the cost matrix with log-normally distributed noise. To create the noise, scaling factors for each entry of the cost matrices *C* were sampled 1000 times from a log-normal distribution with mean μ=0.0 and sigma σ of 0.2,0.5,1.0, and 1.5 . Both tests addressing ranking stability were performed with models trained on the default data split proposed on Kaggle.

#### Test Scenario #2

The second scenario was used to demonstrate the dependence of the outcomes on particular entries in the modified cost matrix. We systematically varied the relationship between the positive and negative cases for each possible confusion, that is, each possible misidentification of classes. We systematically applied a factor *c* in the range between 0.1 and 10 to these cases in the reciprocal form ${\hat{c}}_{\tilde{i},\tilde{\;j}}=c$ , ${\hat{c}}_{\tilde{j},\tilde{\;i}}=\frac{1}{c}$ for the respective selected confusions ( $\tilde{i},\tilde{\;j}$ ). All other entries in the modified cost matrix were set to ${\hat{c}}_{\tilde{i},\tilde{\;j}}=1\;$ for i≠j and ${\hat{c}}_{\tilde{i},\tilde{\;j}}=0$ for i=j .

Based on this, three different modified cost matrices ${\hat{C}}_{\left[\tilde{i},\tilde{\;j}\right]}(c)$ for the particular confusions $\tilde{i},\tilde{\;j}$ selected for systematic variation of weight parameters were given as follows:

$${\hat{C}}_{\left[1,2\right]}(c)=\left(\begin{array}{ccc} 0 & c & 1\\ 1/c & 0 & 1\\ 1 & 1 & 0 \end{array}\right)\;$$

$${\hat{C}}_{\left[1,3\right]}(c)=\left(\begin{array}{ccc} 0 & 1 & c\\ 1 & 0 & 1\\ 1/c & 1 & 0 \end{array}\right)$$

$${\hat{C}}_{\left[2,3\right]}(c)=\left(\begin{array}{ccc} 0 & 1 & 1\\ 1 & 0 & c\\ 1 & 1/c & 0 \end{array}\right)$$

Note that no separate matrices had to be considered in which the costs for positive and negative cases were exchanged, as we varied the values for *c* over the range [0.1,10] . The results for the variation of these cost matrices for *c* in the range between 0.1 and 10 were provided graphically.

In this scenario, we did not analyze the concrete clinical impact. In principle, such a scenario can be related to other clinical settings that includes varying prevalences and severities according to the corresponding use context and specific clinical measures to address the associated risks. Instead, we basically used this scenario to demonstrate the dependence of *WBA_n_* on specific parameters.

## Results

### Basic results of training the ML models

This section presents the results of the experiments described previously. Regarding standard metrics, the VGG16 model trained on the default split of the chest X-ray dataset achieved an accuracy of ∼0.72 in the Test Scenario #1. The other models both achieved an accuracy of ∼0.77 . The confusion matrices visualizing the $a_{i,j}$ values per model when applied on the test dataset are shown in Figure 1.

**Figure 1.**
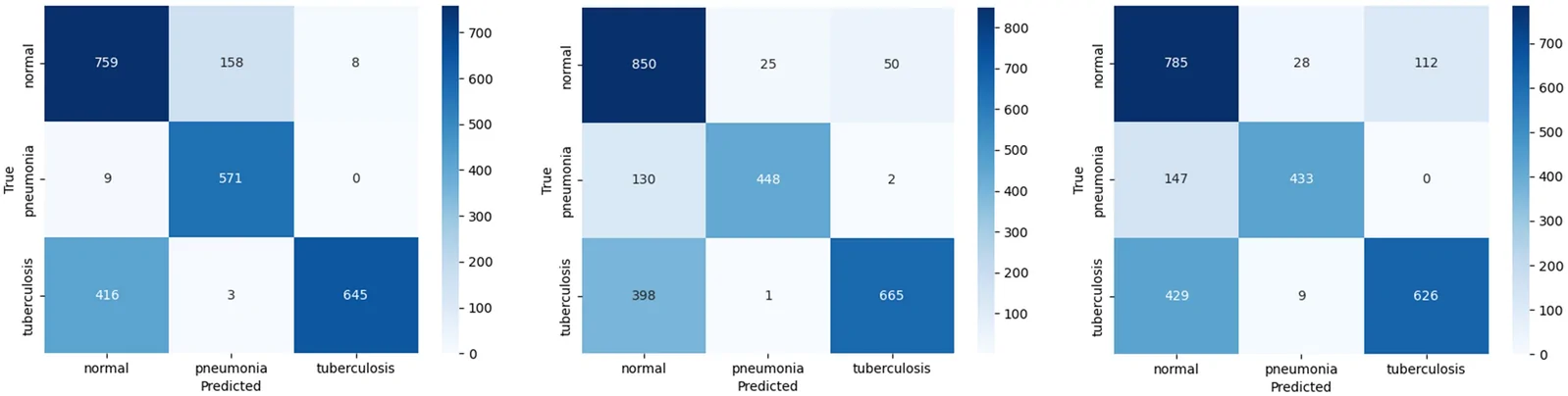
Confusion matrix for the ResNet18 model (left), ResNeXt50 model (middle), and VGG16 model (right) trained on an X-ray dataset.

In the confusion matrix, the value of each field corresponds to $a_{i,j}$ with the classes ordered as 0, normal; 1, pneumonia; and 2, tuberculosis. For example, the second field in the upper row of the confusion matrix for the ResNet18 model ( 158 ) indicates that 158 samples from the test dataset were classified as pneumonia (1) by the model, although they belonged to the normal class (0). From the confusion matrices, the class-conditional probabilities $p_{i,j}$ were calculated according to Equation 1.

Based on these confusion matrices, we first had to calculate $WBA_{n}$ for the given test scenarios, including a derivation of the maximum $ER_{max}$ according to Equation 8 in each case. As a second step, the concrete results for the weighted metrics could be obtained.

### Evaluation of weighted metrics in Test Scenario #1

In Scenario #1, that is, for ${\hat{C}}_{\left[1\right]}$ , the maximum ER for the default data split provided on Kaggle was $ER_{max}=9+10+10=29$ , that is, the sum of the maximum risk in each row of the modified cost matrix ${\hat{C}}_{\left[1\right]}$ .

Table 5 shows the results obtained with 10-fold cross-validation when applying the different weighted metrics, that is, $WBA_{n}$ , CWA , AC , and $\kappa_{w}$ , as well as accuracy as a standard metric to the resulting models. The calculation of all metrics is based on the modified cost matrix ${\hat{C}}_{\left[1\right]}.$ For $WBA_{n}$ , the mean and corresponding 95% CIs across folds are listed for each model.

**Table 5.** ${\mathrm{WBA}}_{n}$ and other metrics for different models in three-class classification. Different metrics are based on identical predictions. ${\mathrm{WBA}}_{n}$ for ResNet18 is highest (approximately 0.85), whereas ${\mathrm{WBA}}_{n}$ for VGG16 is lowest (approximately 0.73). All metrics rank the models identically.

Model	$WBA_{n}$ Mean [95% CI]	CWA Mean [95% CI]	AC Mean [95% CI]	$\kappa_{w}$ Mean [95% CI]	Accuracy Mean [95% CI]
VGG16	0.733 [0.711, 0.756]	0.621 [0.609, 0.638]	2.568 [2.511, 2.620]	0.613 [0.594, 0.641]	0.727 [0.718, 0.740]
ResNet18	0.853 [0.849, 0.855]	0.786 [0.781, 0.790]	1.424 [1.399, 1.458]	0.870 [0.860, 0.878]	0.773 [0.767, 0.781]
ResNeXt50	0.777 [0.753, 0.798]	0.673 [0.655, 0.694]	2.177 [2.082, 2.209]	0.704 [0.687, 0.725]	0.769 [0.761, 0.776]

CI: confidence interval.

Table 5 shows that ResNet18 model achieves the highest $WBA_{n}\;(∼0.85\pm 0.01$ ). For all models, $WBA_{n}$ is slightly higher than their corresponding accuracy. In this scenario, the other metrics lead to an identical ranking of the models.

The evaluation of ranking stability across different subsets of the test dataset, assessed via bootstrap sampling, is documented in Table 6. It can be seen from Table 6 that the ResNet18 model was ranked as the best model by all weighted metrics in 100% of cases. In contrast, standard accuracy ranked the ResNet18 model as the best model in only 72.0% of cases. The metric also ranked the ResNeXt50 model as the best model in 25.6% of cases and the VGG16 model in 2% of cases.

**Table 6.** Probability that a given model is ranked as the best model by different metrics when drawing 1000 bootstrap samples of size 2500 from the test data set.

Metric	VGG16	ResNet18	ResNeXt50
Accuracy	0.024	0.720	0.256
$WBA_{n}$	0.000	1.000	0.000
CWA	0.000	1.000	0.000
AC	0.000	1.000	0.000
$\kappa_{w}$	0.000	1.000	0.000

The evaluation of ranking stability across different cost matrices, assessed using cost matrices sampled with log-normally distributed noise, is documented in Table 7. It can be seen from Table 7 that CWA , AC and $WBA_{n}$ lead to an identical ranking of the models for any value of *σ*. When setting σ to 0.2 and 0.5 , respectively, the metrics ranked the ResNet18 model as the best model in 100% of cases. When setting σ to 1.0 , the metrics ranked the ResNet18 model as the best model in 97.5% of cases and the ResNeXt50 model as the best model in the remaining 2.5% of cases. In the case of σ=1.5 , the ResNeXt50 model was ranked as the best model in 10.4% of cases. The VGG16 model was never ranked as the best model by these metrics. In the case of $\kappa_{w}$ , the ranking was slightly different. The ResNeXt50 model was ranked as the best model in 3.1% of cases when setting σ to 0.5 . When setting σ to 1.0 and 1.5 , this value increased to 16.1% and 24.5% , respectively. In the case of σ=1.5 , the VGG16 model also had a 0.1% probability of being selected as the best model.

**Table 7.** Probability that a given model is ranked as the best model by different metrics when sampling 1000 cost matrices C with log-normally distributed noise.

Metric	σ	VGG16	ResNet18	ResNeXt50
$WBA_{n}$	0.2	0.000	1.000	0.000
	0.5	0.000	1.000	0.000
	1.0	0.000	0.975	0.025
	1.5	0.001	0.895	0.104
CWA	0.2	0.000	1.000	0.000
	0.5	0.000	1.000	0.000
	1.0	0.000	0.975	0.025
	1.5	0.001	0.895	0.104
AC	0.2	0.000	1.000	0.000
	0.5	0.000	1.000	0.000
	1.0	0.000	0.975	0.025
	1.5	0.001	0.895	0.104
$\kappa_{w}$	0.2	0.000	1.000	0.000
	0.5	0.000	0.969	0.031
	1.0	0.001	0.893	0.161
	1.5	0.000	0.755	0.245

### Evaluation of Test Scenario #2

In the second scenario, that is, in the case of ${\hat{C}}_{\left[\tilde{i},\tilde{\;j}\right]}(c$ ), the maximum $ER_{max}(c)$ depended on the value of *c* and the selected confusion ( $\tilde{i},\tilde{\;j}$ ) that was used for the modification of weights. In the cases with c≥1 , $ER_{max}(c)$ had to be calculated as $ER_{max}(c)=c+1+1$ . For c<1 , this calculation had to be adapted to $ER_{max}=1+\frac{1}{c}+1$ .

This applied to all cases of confusion ( $\tilde{i},\tilde{\;j}$ ) selected for the systematic modification of the weight parameters. Figure 2 shows how $WBA_{n}$ changes when varying *c* between 0.1 and 10 (using a logarithmic scale).

**Figure 2.**
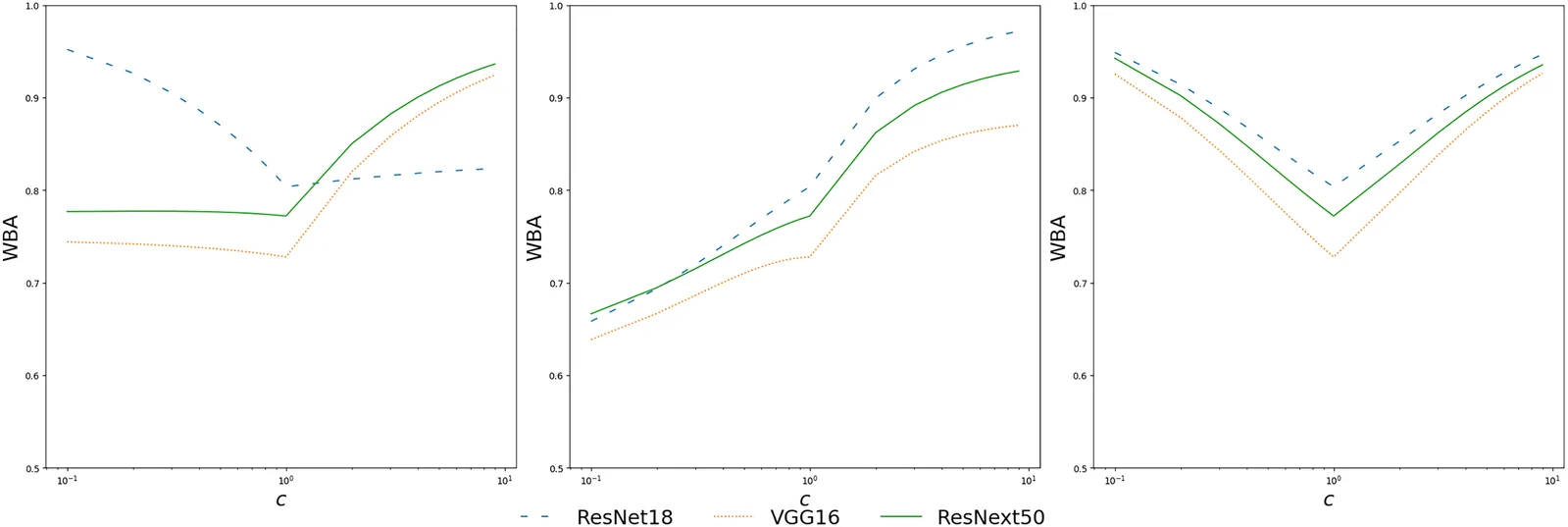
Changes in ${\mathrm{WBA}}_{n}$ for different ML models when varying c in ${\hat{C}}_{\left[1,2\right]}$ (left), ${\hat{C}}_{\left[1,3\right]}$ (middle), and ${\hat{C}}_{\left[2,3\right]}$ (right) between 0.1 and 10 (using a logarithmic scaling for c ); the course of ${\mathrm{WBA}}_{n}$ differs substantially between c<1 and c>1 : ${\hat{C}}_{\left[1,2\right]}$ : decrease for ResNet18 when increasing c from 0 to 1 whereas VGG16 and ResNeXt50 remain fairly constant; when c increases from 1 to 10 , ${\mathrm{WBA}}_{n}$ increases continuously, especially for VGG16 and ResNeXt50; the ordering of ML models changes in this area, that is, ResNet18 model becomes the worst model when setting c>2 . ${\hat{C}}_{\left[1,3\right]}$ : increase in all models over the entire range of c ; no impact on ordering of models, except a minor switch between ResNet18 and ResNeXt50 for low values of c . ${\hat{C}}_{\left[2,3\right]}$ : decrease in all models for c<1 ; increase in all models for c>1 ; no impact on ordering of models.

ML: machine learning; WBA: weighted balanced accuracy.

It can be seen from Figure 2 that the behavior of $WBA_{n}$ differs substantially between the regions c<1 and c>1 . This is caused by a discontinuity resulting from the change in the calculation of $ER_{max}(c)$ from $ER_{max}(c)=1+\frac{1}{c}+1\;$ to $ER_{max}(c)=c+1+1$ at c=1 . In particular, this is due to the switch of the cost-maximizing class *j*^∗^(*i*), which results in a nondifferentiable point in $ER_{max}(c)$ at *c*=1.

For example, in the case of ${\hat{C}}_{\left[1,2\right]},\;WBA_{n}$ of ResNet18 systematically decreases as c<1 changes. However, ResNet18 remains the best ML model in this region because it achieves higher $WBA_{n}$ values, above the 0.8 level. The other models, that is, VGG16 and ResNeXt50, remain relatively constant at a level below 0.8 in this region. This behavior is reversed to some extent for c>1 . Here, the $WBA_{n}$ values for VGG16 and ResNeXt50 continuously increase, whereas those for ResNet18 remain relatively constant. Consequently, the ranking of model performance changes in this region. In the region c<1 and slightly beyond, ResNet18 remains the best model because it achieves higher $WBA_{n}$ values than VGG16 and ResNeXt50. Starting at approximately c∼2 , however, the other models take the lead, further increasing their performance advantage over ResNet18. Regarding the comparison between VGG16 and ResNeXt50, ResNeXt50 maintains higher performance across the entire range from 0.1 to 10.0.

For ${\hat{C}}_{\left[1,3\right]}$ , $WBA_{n}$ increases across all models over the entire range of *c*. No change in model ranking is observed, except for a minor switch between ResNet18 and ResNeXt50 at c∼0.25 . For ${\hat{C}}_{\left[2,3\right]}$ , $WBA_{n}$ decreases across all models for c<1 and increases across all models for c>1 . Again, no change in model ranking is observed.

These effects are related to the class-specific performance of each model, as visualized by the confusion matrices in Figure 1. For example, the (*FP*) and (*FN*) cases for pneumonia, that is, confusion $\left(\tilde{i},\tilde{\;j})=(1,2\right)$ , show opposing trends between VGG16 and ResNeXt50 on the one hand and ResNet18 on the other. This is reflected in the corresponding diagrams on the left side of Figure 2.

The curves are almost mirror images for VGG16/ResNeXt50 and ResNet18 with respect to the line c=1. For the confusion $\left(\tilde{i},\tilde{\;j})=(1,3\right)$ , shown in the middle of Figure 2, the curves follow the same direction, reflecting the asymmetry in the distributions of (*FP*) and (*FN*) cases for tuberculosis across all trained models. For $(\tilde{i},\tilde{\;j})=(2,3),$ all models have only a limited number of actual confusions between tuberculosis and pneumonia, resulting in a dominance of the other classification cases. Consequently, the contribution of the other errors becomes less influential as the value deviates from c=1 . This leads to an increase in the $WBA_{n}$ values toward both ends of the diagram, as shown on the right side of Figure 2.

## Discussion

In this study, we developed $WBA_{n}$ as a risk-based approach for assessing multiclass classification models in medical applications. The definition of $WBA_{n}$ was designed to operationalize the regulatory requirements for medical devices established by the relevant legislation and standards in the EU. These include the MDR,^8^ ISO 14971,^10^ and ISO/TR 24971,^12^ which provide corresponding requirements and guidance for RM. The proposed $WBA_{n}$ metric extends the WBA metric^11^ to multiclass settings. We demonstrated the application of $WBA_{n}$ in a concrete medical use case using a reference database for the detection of lung diseases from X-ray images.

### Relation to basic regulatory requirements

For medical devices, the MDR^8^ and ISO 14971^10^ require the implementation of an RM process that systematically reduces risks as far as possible. Similar requirements apply under the EU AI Act^9^ to so-called high-risk AI systems. Because ML-based medical devices generally fall within the scope of high-risk AI systems under Article 6 of the AI Act, they are also subject to corresponding RM obligations.^9^ The MDR and AI Act share basic principles with respect to RM requirements but they also differ in certain details. For example, the MDR permits risk-control measures to be limited when no further improvement in the risk-benefit ratio can be achieved.^8^ However, the fundamental approach to RM remains consistent across these frameworks. This alignment particularly supports the risk-based assessment approach proposed in this study.

This includes defining risk as a combination of the likelihood and severity of a potential harm.^8,9^ For ML-based classification models, this means that the impact of different types of errors must be appropriately considered when assessing model performance. Pure error-based measures, as used in many standard metrics such as accuracy, F1 score, or PPV, are insufficient for this purpose. When the impact of risks is incorporated into an appropriate metric, both likelihood and severity must be quantified accordingly. Regarding the assessment of risk factors, ISO 14971^10^ and ISO/TR 24971^12^ permit a semi-quantitative approach in which different levels of likelihood and/or severity are classified into a discrete set of categories. As proposed in ISO/TR 24971,^12^ likelihood levels are often separated by a factor of 10. Additionally, ISO/TR 24971^12^ describes a multiplicative combination of likelihood and severity as a basis for defining the overall risk level. When the resulting risk levels remain constant along the diagonals of the risk matrix—that is, when a one-level decrease in likelihood compensates for a one-level increase in severity and vice versa—the costs between two adjacent severity levels also differ by a factor of 10 .

The $WBA_{n}$ approach presented in this study integrates these basic components into a single metric for a consistent risk-based assessment of ML models, designed to operationalize the framework required by ISO 14971.^10^ It is based on the concept of ER and enables the overall level of risk to be assessed according to these relationships. This approach is consistent with the requirements of both the MDR^8^ and the AI Act.^9^

Additionally, applicable regulatory requirements require RM to be systematically integrated throughout the entire product lifecycle.^8–10^ Accordingly, RM needs to be incorporated into the development of ML models, although most scientific publications on ML-based models for medical applications do not adopt such an approach.^11^ This obligation cannot be deferred solely to the stage of clinical translation under the aforementioned requirements. In particular, this applies to ML models performing multiclass classification tasks. In such cases, a purely threshold-based approach is not feasible, precluding risk adjustment through threshold adaptation according to the specific medical scenario. Consequently, multiclass classification generally requires more extensive integration of risk considerations into the model-development process.

In principle, the risk-based weighting incorporated into the $WBA_{n}$ can be integrated into different stages of the development pipeline, including both pre- and postprocessing steps. For example, $WBA_{n}$ could be incorporated into model training and validation/tuning, although the practical applications presented in this study were limited to model assessment.

### Comparison of ${WBA}_{n}$ with existing weighted metrics

For binary classification tasks, a risk-based approach to assessing ML models was previously introduced.^11^ The WBA metric proposed in that study uses risk-based weighting that represents a multiplicative combination of the probability and severity of the corresponding risks. This enables the impact of different types of errors, such as (*FP*) and (*FN*) classifications, to be systematically incorporated into the assessment of an ML model. However, the previously published WBA approach^11^ is limited to binary classification tasks. The $WBA_{n}$ metric proposed in the present study systematically extends the WBA metric to multiclass settings. Thus, WBA represents a special case of $WBA_{n}$ for *n* = 2.

Other weighted metrics, such as average cost (AC), ^18^ weighted kappa score $\kappa_{w},$ ^25^ and cost-weighted accuracy ( CWA ),^26^ can also be applied to multiclass settings. These metrics allow specific costs or utilities to be assigned to particular combinations of actual and predicted classes.

The results obtained in Scenario #1 showed that the different weighted metrics, particularly CWA and AC , yielded similar results and an identical model ranking to $WBA_{n}$ . However, these metrics do not provide a consistent implementation of risks. Specifically, they do not systematically incorporate the likelihood and severity of potential harm as the fundamental components of risk. To address this limitation, we replicated the construction of the weighting matrices for AC , $\kappa_{w}$ , and CWA by using the cost matrix from the $WBA_{n}$ approach. However, this does not constitute a systematic risk-based methodology; rather, it represents a pragmatic compromise that enables comparison of the basic outcomes across metrics using the same cost matrix. Only in the $WBA_{n}$ approach could the construction of the cost matrix be systematically operationalized according to the regulatory requirements of ISO 14971^10^ and ISO/TR 24971.^12^ This was not possible for the other weighted metrics AC , $\kappa_{w}$ , and *CWA*.

A particular issue with metrics such as AC , CWA and $\kappa_{w}$ is that the costs incorporated into these metrics are not systematically related to the real-world scenario in which the ML model is ultimately applied. This is particularly important when the class distribution differs substantially between the real-world and development settings. The latter refers to the distribution of actual classes in the training, validation/tuning, and test datasets. In such situations, a cost matrix may be defined for the intended use case and applied indiscriminately to both the development and real-world settings, potentially resulting in nonrepresentative risk estimates for one or both settings. In contrast, $WBA_{n}$ systematically incorporates the relationship between the class distributions in the real-world setting $d_{i}$ into the definition of the metric. Additionally, the probabilities derived from the development datasets are incorporated into the calculation of the class-conditional probabilities $p_{i,j.}$ In summary, $WBA_{n}$ systematically establishes the required relationship between these different settings. This is achieved by incorporating the class distributions of the real-world setting $d_{i}$ into the modified cost matrix ${\hat{C}}_{\left[i,j\right]}$ .

Without such an adaptation, weighted metrics may be affected by class imbalance or, more precisely, by an inappropriate balance between classes.^14^ In particular, this applies to AC , $\kappa_{w},$ and CWA , which do not systematically adjust for real-world class distributions. This represents a major challenge in medical applications, particularly in diagnostic tasks, where a substantial imbalance often exists between positive and negative cases, that is, cases with and without disease.^14^ On the one hand, a balanced class distribution is typically pursued when constructing development datasets to achieve comparable robustness across classes. In contrast, the relationship between the development and real-world class distributions must be considered to achieve an appropriate balance of costs in the metrics.

Sometimes, the higher severity of a particular type of error, for example, FP , may be approximately offset by the lower likelihood of this error in the real-world setting. Consequently, the effect of inadequately weighting risks may not be immediately apparent in some scenarios. However, such a balance should not occur merely by chance; rather, risks should be systematically incorporated into the assessment methods.

Furthermore, $WBA_{n}$ yields a normalized score ranging from 0 to 1, enabling comparisons across different use cases. This is particularly useful when comparing the performance of different models, devices, datasets, or use cases. Metrics such as AC or $\kappa_{w}$ scale with the magnitude of the costs and therefore cannot be directly compared across different settings. CWA also incorporates normalization; however, it uses an inactive classifier as the reference. This scaling does not provide a robust basis for comparing different models. In particular, it becomes problematic when a model performs worse than the aforementioned inactive classifier, which may occur in settings with highly imbalanced costs or class distributions.

Based on these observations, $WBA_{n}$ offers several advantages over existing approaches. Table 8 summarizes the key characteristics of these metrics.

**Table 8.** Comparison of different weighted evaluation metrics; ${\mathrm{WBA}}_{n}$ is directly related to risk, generates normalized scores, and is applicable to multiclass classification.

Criterion	AC	$\kappa_{w}$	CWA	WBA	$WBA_{n}$
Multiclass classification	Yes	Yes	Yes	No	Yes
Designed to operationalize risk definition (as combination of likelihood and severity)	Indirectly	No	No	Yes	Yes
Consequent relation to risk	Yes	No	No	Yes	Yes
Alignment to class distributions	Yes	No	No	Indirectly	Yes
Robust toward class imbalance	No	No	No	Yes	Yes
Normalized	No	No	Yes	Yes	Yes
Comparable among use cases	No	No	No	Yes	Yes
Applicable for thresholding	No	No	No	Yes	No

### Practical application of WBA_n_

To demonstrate the practical applicability of the $WBA_{n}$ metric, we used an existing dataset representing the diagnosis of lung diseases. The dataset had several limitations, most notably that class membership was confounded with patient population and acquisition protocol. However, these limitations did not affect the demonstration of the basic applicability of $WBA_{n}$ , which was the primary objective of this study. Nevertheless, they limited the clinical interpretability of the absolute model performance. A more rigorous approach would therefore be required when translating the metric into an actual clinical application.

For this specific scenario, we first had to assess the likelihood and severity of the potential risks. This is a critical step because these assessments provide the basis for calculating the corresponding weights. In principle, the likelihoods are closely related to the prevalences observed in the intended medical application, that is, the real-world setting. In this study, we derived these prevalence estimates from the literature, although some deviations and uncertainties remained. In other settings, particularly for new devices or applications, these estimates may need to be obtained from alternative data sources or dedicated studies.

It should be noted that prevalence may vary substantially across different real-world scenarios, even when the underlying diagnostic task is the same. For example, disease prevalence is generally substantially higher when a diagnostic test is applied to patients with a specific clinical suspicion than when it is used as part of a general screening procedure. Similar dependencies on the real-world setting may apply to both likelihood and severity assessments. The assessment of severity may depend, in particular, on the measures available for managing the associated risks. For example, the availability of a second diagnostic test or an appropriate treatment option may influence the severity assigned to a given outcome. In this study, severity was assessed only semi-quantitatively, based on a rough estimation of the potential impact of the respective outcomes.

Overall, the comparison of the different ML models was relatively consistent across the weighted metrics, particularly in Test Scenario #1. Importantly, all metrics were calculated using a cost matrix ${\hat{C}}_{1}$ that already incorporated weighting based on the real-world class distribution. However, when metrics such as AC , CWA , or $\kappa_{w}$ are applied without accounting for real-world class distributions, the results may differ and potentially be less representative. Test Scenario #2 further demonstrated that changes in the risks or weights represented in the cost matrix can lead to substantial differences in model evaluation.

For example, such changes may alter the ranking of model performance and, consequently, lead to the selection of a different ML model as the preferred alternative when inappropriate weighting or no weighting is used.

In the practical applications examined in this study, these effects remained limited. This was primarily because the models had relatively similar confusion matrices and the same cost matrices derived using the $WBA_{n}$ approach were applied to all models. In these applications, the calculation of $WBA_{n}$ was therefore primarily determined by the confusion matrices, as the metric was used only for assessment purposes. This situation may differ when weighted metrics are more deeply integrated into the model-development process, for example, when weights are incorporated during training or validation/tuning in the context of CSL.

The generalized multiclass $WBA_{n}$ behaves intuitively by penalizing models that perform poorly on high-risk error types. Importantly, model rankings based on $WBA_{n}$ can differ substantially from those based on classical accuracy. For example, although ResNet18 achieved the highest accuracy, VGG16 and ResNeXt50 outperformed ResNet18 under certain cost structures (Figure 2). This demonstrates that classical metrics may not adequately reflect model performance in high-stakes settings and highlights the value of risk-based metrics for meaningful evaluation.

### Limitations

Despite its advantages, the $WBA_{n}$ approach and the analysis presented in this study have several limitations. First, the definition of the cost matrix depends on the accuracy of the assessments of risk likelihood and severity. Consequently, uncertainties or inaccuracies in these assessments are propagated to the final scores or values of the $WBA_{n}$ metric. This limitation generally applies to weighted metrics that rely on assessments of risks or other costs associated with the intended clinical application. For $WBA_{n}$ , however, the derivation of costs is grounded in a systematic, risk-based approach. For other metrics, the assignment of costs may be more arbitrary.

In this study, we used a semi-quantitative approach to define likelihood and severity according to ISO 14971^10^ and ISO/TR 24971.^12^ We used available literature and plausible assumptions to derive appropriate ratings; however, we could not fully capture all aspects of the intended use case. Ideally, these ratings would be derived from an empirically and clinically validated risk model based on input from relevant domain experts, clinical outcome data, or structured elicitation methods. Such a model should also characterize the associated uncertainties. Because $WBA_{n}$ is explicitly cost-sensitive, inappropriate or subjective cost definitions may systematically bias model rankings and potentially lead to an underestimation of real-world risk.

Regarding risk estimation and the subsequent derivation of the cost matrix, our study did not achieve a fully comprehensive or formally validated assessment because an expert panel was not available to validate the estimations. The primary aim of this study was to demonstrate the overall process for applying the $WBA_{n}$ metric. As described above, this process can be implemented using a semi-quantitative approach based on expert consensus. In practice, however, such an approach remains challenging and inherently subjective to some extent. Nevertheless, disregarding potential risks because precise information is unavailable is not an appropriate alternative, as doing so could further compromise the risk assessment and development of medical devices. Accordingly, ISO 14971 and ISO/TR 24971 allow the use of expert panels to support semi-quantitative risk assessment when precise quantitative data are unavailable.

Furthermore, the current formulation of $WBA_{n}$ does not support multilabel classification, hierarchical label structures, or sequential decision-making tasks. Moreover, $WBA_{n}$ does account for epistemic uncertainty or the risk associated with out-of-distribution samples, both of which are increasingly relevant to clinical AI systems and may substantially affect the likelihood of FN or FP predictions.

Regarding practical application, we evaluated the approach in only one medical use case using an existing Kaggle dataset. We did not perform dedicated data collection for the specific real-world scenario under consideration, nor did we conduct a prospective or targeted clinical study. Instead, the Kaggle dataset was retrospectively evaluated as a cohort of individuals. The dataset also had limitations in terms of the availability of detailed demographic information. Furthermore, the pathologies were not equally distributed across the dataset because the constituent datasets originated from different sources. These components also differed in important characteristics, including patient age and the pathologies represented; for example, some datasets predominantly comprised pediatric cases, whereas others primarily comprised adults. The individual datasets were amalgamated to create a multiclass dataset representing the overall use case. Consequently, the resulting dataset should be regarded primarily as a proof-of-concept dataset rather than as a representative sample of a specific clinical population.

We included a basic comparison of the different risk-based metrics without assessing their overall validity for the specific use case. To obtain more robust estimates, we performed k-fold cross-validation and a sensitivity analysis using log-normal scaling of the cost matrix. Additionally, we examined the stability of model rankings across different population subgroups using bootstrap sampling. However, these analyses do not establish how the approach would perform in other clinical scenarios. Rather, they illustrate the process of implementing the $WBA_{n}$ approach and demonstrate its impact in the specific scenario examined in this study. This is consistent with the scope of the study, which primarily aims to introduce a normalized metric for multiclass classification that aligns with the regulatory definition of risk as a combination of probability and severity. The study was not intended to comprehensively establish the validity of the $WBA_{n}$ approach across different clinical use cases. More generally, its validity and suitability should be evaluated and justified on a case-by-case basis for the specific application and clinical context.

Notably, we applied the $WBA_{n}$ approach only to the assessment of the ML models and not to other stages of model development, such as CSL-based training. The latter could enable the development process to be guided toward an ML model that explicitly minimizes the relevant risks and may therefore provide a stronger demonstration of the impact of the $WBA_{n}$ approach. More broadly, comprehensive risk reduction requires risk-based considerations to be incorporated throughout the model-development process rather than only during final assessment. Furthermore, multiclass classification tasks generally do not offer the same threshold-based postprocessing options available in binary classification. In binary classification, clinicians can adjust the decision threshold to adapt the model to specific clinical settings and risk preferences. For multiclass classification, such straightforward threshold adjustment is generally not available, further emphasizing the importance of systematically incorporating risk assessments into model development.

However, the robustness of CSL-based ML model training using risk-based approaches requires careful evaluation. Weighted metrics may be more susceptible to instability or unintended effects during model optimization, and these potential issues should therefore be systematically assessed. This should include a more detailed evaluation of the calibration properties of the resulting models, which was beyond the scope of the present study.

### Implications for future work

Overall, $WBA_{n}$ provides a promising foundation for the risk-based evaluation of ML models. However, its practical utility currently depends on well-defined cost assessments, robust statistical evaluation, appropriately calibrated models, and further development of its theoretical foundations. Future work should therefore investigate the integration of $WBA_{n}$ with uncertainty quantification and calibration methods. This could provide more reliable estimates of the probabilities of correct and incorrect classifications, leading to more reliable risk scores and potentially supporting more effective RM activities. Another important research direction is the extension of $WBA_{n}$ to multilabel classification, hierarchical label structures, and sequential decision-making tasks.

Furthermore, the approach should be evaluated across a broader range of medical applications and clinical use cases. This study provides an initial proof of concept focused on demonstrating the practical applicability of the approach rather than comprehensively validating it. Future research should therefore apply the $WBA_{n}$ approach a range of well-defined clinical scenarios to assess its validity, robustness, and generalizability across different settings.

Additionally, we used the risk-based approach only for model assessment and did not apply the $WBA_{n}$ metric during other stages of model development, such as ML model training using a CSL approach. Further research in this direction could determine the extent to which incorporating risk-based optimization during model development can improve model performance in terms of risk reduction and clinical impact.

## Conclusion

In this study, we developed $WBA_{n}$ as a risk-based approach for assessing ML models that perform multiclass classification. We demonstrated its application in concrete medical scenarios and designed the approach to operationalize the RM requirements for medical devices defined by the EU MDR^8^ and the corresponding RM standards.^10,12^ The approach incorporates several core principles:

-
$WBA_{n}$ is a weighted metric in which the weights represent a systematic assessment of risks.^8,10,12^ In this context, risk is defined as the multiplicative combination of likelihood and severity for a particular application. The assessment of individual risks is linked to the corresponding types of prediction errors in the classification task. Accordingly, optimization of $WBA_{n}$ corresponds to minimizing the ER associated with potential misclassifications.
- The definition of $WBA_{n}$ systematically accounts for the relationship between the development and real-world settings. Specifically, it considers the class distributions in the development datasets used for training, validation/tuning, and testing in relation to the class distribution in the intended medical application. This highlights the importance of thoroughly analyzing these distributions to appropriately balance the risks associated with different types of classification errors in a specific medical setting.
- The application of $WBA_{n}$ can be based on either quantitative or semi-quantitative assessments of risk likelihood and severity.^10,12^
-
$WBA_{n}$ extends the WBA approach.^11^ Thus, the risk-based assessment is systematically extended from binary to multiclass classification tasks.
-
$WBA_{n}$ is normalized to the range of 0 to 1 to facilitate direct comparison between different ML models. The normalization is based on the maximum ER for the given application scenario.

In this study, we applied the $WBA_{n}$ approach to a concrete medical use case involving X-ray-based detection of lung diseases. We used a standardized dataset from the Kaggle database as an initial reference dataset and compared different ML models using $WBA_{n}$ and other weighted metrics. Additionally, we analyzed the dependence of the $WBA_{n}$ metric on specific parameters of the weight/cost matrix.

The results demonstrated that the applicability of $WBA_{n}$ approach to a concrete medical use case. In this limited setting, its results were broadly consistent with those obtained using other metrics. However, the other metrics were not systematically grounded in the RM requirements defined for medical devices. Furthermore, the results demonstrated that changes in risk relationships can affect model selection by altering the ranking of models according to their corresponding $WBA_{n}$ values.

Based on these findings, the $WBA_{n}$ approach provides a systematic framework for the development and assessment of ML models for medical applications involving multiclass classification tasks. Several opportunities remain to extend its applicability. For example, in its current form, $WBA_{n}$ cannot be applied to multilabel classification. In addition, this study does not provide an approach for integrating $WBA_{n}$ with uncertainty quantification. Such an extension could enable the systematic estimation of uncertainty in model outputs and its incorporation into risk assessment. Furthermore, the applicability and validity of the proposed approach should be evaluated across a broader range of medical applications and clinical scenarios.

Overall, this study provides a normalized and comparable metric for assessing ML models based on risk rather than solely on error rates. It therefore contributes to the systematic implementation of RM requirements in ML-based medical applications. This is particularly important for ML models incorporated into medical devices, where compliance with the applicable regulatory requirements is essential.

## Supplemental Material

sj-pdf-1-imr-10.1177_03000605261486667 - Supplemental material for Normalized risk-based evaluation of machine learning–based classification models: A multiclass approach with applications in medical devices Supplemental material, sj-pdf-1-imr-10.1177_03000605261486667 for Normalized risk-based evaluation of machine learning–based classification models: A multiclass approach with applications in medical devices by Julius Wiggerthale, Martin Haimerl and Christoph Reich in Journal of International Medical Research

## Data Availability

The application of the method was based on an existing open-access data set that only included fully anonymized data. The dataset is publicly available through Kaggle at https://www.kaggle.com/datasets/muhammadrehan00/chest-xray-dataset. As the study used only existing, fully anonymized data, no additional ethics committee approval or patient consent was applicable.
