# Supplementary material for: Normalized risk-based evaluation of machine learning–based classification models: A multiclass approach with applications in medical devices
Source: J Int Med Res. 2026 Sep 28;54(9):03000605261486667. doi: 10.1177/03000605261486667 (PMC13735062; doi:10.1177/03000605261486667)
Supplement: Supplementary file 1 [file sj-pdf-1-imr-10.1177_03000605261486667.pdf]

STROBE Statement — checklist of items that should be included in reports of observational studies

Wiggerthale J, Haimerl M, Reich C. Normalized Risk-Based Evaluation for Machine-Learning based Classification Models – A Multiclass Approach with Applications in Medical Devices

	Item No.	Recommendation	Page No.	Relevant text from manuscript
**Title and abstract**	1	(*a*) Indicate the study's design with a commonly used term in the title or the abstract	1	Title: "Normalized Risk-Based Evaluation for Machine-Learning based Classification Models – A Multiclass Approach with Applications in Medical Devices"<br><br>Remark: The study design was not directly included since this was not the main focus of the paper. Instead, the title and abstract focus on the development of the concept for the WBA_n metric and its subsequent practical application.
		(*b*) Provide in the abstract an informative and balanced summary of what was done and what was found	1	"METHODS In this paper, we provide Weighted Balanced Accuracy WBA_n as a risk-based approach for the assessment of ML models for multiclass classification. WBA_n is systematically based on regulatory requirements for medical devices. It implements core properties like representation of risks as a multiplicative combination of likelihood and severity, and/or the relationship between development and real-world scenarios. We demonstrate the application of WBA_n using an X-ray based detection of lung diseases as a reference scenario.<br>RESULTS Our analysis shows that WBA_n achieves a risk-based assessment in the lung disease scenario as a reference application. WBA_n systematically operationalizes the definition of risk from corresponding regulatory requirements and allows to adapt the assessment according to risk levels that have to be elaborated in the context of the risk management process.<br>CONCLUSION Even though this paper only used a single use case to demonstrate the practical applicability of our approach, it shows the

				impact of a risk-based approach when assessing the performance of ML multiclass classification models in medical applications. Without such an approach, regulatory requirements cannot be implemented in a fully comprehensive way. Subsequently, the clinical impact cannot be rated consequently when applying the model in real-case scenarios
**Introduction**				
Background/rationale	2	Explain the scientific background and rationale for the investigation being reported	1-3	The background is elaborated in several aspects in the introduction. This includes<br>• the utilization of ML-based approaches in medical applications<br>• basic regulatory requirements when using ML-based techniques in medical devices<br>• the necessity of risk-based assessment techniques integrating the impact of different error types in classification tasks<br>• stages of application in the development chain, including cost-sensitive learning using direct and meta-learning approaches<br>• already existing weighted metrics, e.g. average cost, weighted kappa score, or cost-weighted accuracy<br>• weighted balanced accuracy as a systematic risk-based approach for the binary case<br>Further information is included in the Materials and Methods section, since this more detailed material was required to systematically develop the WBA_n approach based on existing frameworks.
Objectives	3	State specific objectives, including any prespecified hypotheses	3	The follow text at the end of the introduction is included:<br>“To address these limitations, the main objectives of this paper are the extension of the WBA metric to a multiclass setting and the demonstration of its applicability as well as its impact in practical applications. In other

words, we elaborated the WBA_n metric as a normalized metric that is designed to operationalize the structure required by [10]. Within our approach, we make use of a multi-dimensional cost matrix that represents the different class constellations and consequently follows regulatory requirements regarding RM for medical devices. Based on these goals, we define WBA_n as a risk-based metric that retains the desirable properties of the binary formulation while becoming applicable to arbitrary numbers of classes. This includes explicit relation to expected risk, systematic integration of class distributions / imbalance, and normalization to values between 0 and 1 for allowing a direct comparison between different ML models.

To exemplarily illustrate the practical applicability, we apply the approach to the training and assessment of ML models a concrete use case addressing a multi-class classification task. For this purpose, the detection of lung diseases in x-ray images was utilized. An open access data set from the Kaggle database was used. In principle, this represents a retrospective observational / cross-sectional study. However, the study itself is not the main objective of the paper. Instead, it aims at demonstrating the application of the risk-based methodology to this particular exemplary scenario. This includes a comparison of the results based on the the WBA_n metric to already existing weighted metrics. Additionally, the impact regarding changes with respect to the weights in the cost matrix is demonstrated. Furthermore, the conformance with regulatory requirements for risk management in the field of medical devices is getting assessed.

Our analysis only focuses on the evaluation of ML models. It does not include the inclusion of risk-based metrics into the training of the models like the mentioned CSL approaches. Even though this should be considered as an extension towards a fully comprehensive integration of

				risk-based approaches into the development of ML-based medical devices, this was beyond the scope of this paper.
**Methods**				
Study design	4	Present key elements of study design early in the paper	7-10	see Subsection "Experimental Setup to Demonstrate the Application of WBA_n"<br><br>Basically, the study is a retrospective observational / cross-sectional study based on an existing Kaggle data set. Thus, the paper does not include an own dedicated study. Instead, the purpose of this analysis is the demonstration of the practical applicability of WBA_n. Based on this, also the following categories do only apply in a limited way.<br><br>In the paper, the following statement s included:<br><br>"To demonstrate how WBA_n applies to real world applications, we conducted experiments using a specific medical application scenario. This study did not involve any new acquisition of data from human participants. It relied exclusively on a publicly available, fully anonymized dataset that does not permit re-identification of individuals. Such secondary use of anonymized, publicly available data does not constitute research on identifiable human subjects. Consequently, ethics committee approval and informed consent were not required. Additionally, the previously published data sets were performed according to the Declaration of Helsinki, performed under IRB approval and/or explicitly exempted from IRB review [27, 28, 29, 30]. Based on this, our analysis was conducted in accordance with the principles of the Declaration of Helsinki based on the rules regarding secondary use of anonymized data therein.<br><br>We utilized the chest X-ray dataset for the diagnosis of lung diseases that is available on Kaggle under https://www.kaggle.com/datasets/muhammadrehan00/chest-xray-dataset [311]. Formally, this represents a retrospective observational / cross-sectional study. No specific hypothesis was addressed regarding the concrete results for the used metrics. Instead, this example served to illustrate the practical applicability of the WBA_n

metric and compare it to other weighted evaluation metrics. This demonstrates the steps that have to be performed to set up the cost matrix and apply the WBA_n metric to a concrete use case. This addresses the main objective of the paper, i.e. the definition of the WBA_n metric as a normalized metric that is designed to operationalize the structure required by [10]. Regarding the study design and data preparation we did not perform additional steps. We basically took the existing data set as it was provided. Thus, there was no specific reporting about missing data and also no specific measure to deal with missing data. Within the particular data sets, there were no missing data since only the X-ray images were used. No specific measures for loss to follow-up were be applied. No specific subgroup analysis was performed. No dedicated analysis of the cases in the different steps of the case selection was provided that e.g. could be provided in a flow diagram. Thus, also no particular reasons for for non-participation at each stage had to be reported. In general, the reporting of this study conforms to STROBE guidelines. To demonstrate the impact of a risk-based assessment based on the WBA_n, the analysis in this paper includes a comparison with existing metrics. Additionally, the dependency of the WBA_n metric on specific parameters of the (modified) cost matrix was analyzed. Furthermore, a sensitivity analysis was performed to demonstrate the robustness of the approach. Based on this, the provided analysis represents a first proof-of-concept regarding the WBA_n approach and does not provide a concise analysis of the outcomes for the particular medical scenario used as a reference.

The used data set consisted of X-ray images belonging to the classes normal (N), pneumonia (P), and tuberculosis (T). It was divided into a predefined training, validation/tuning, and a test data set. An overview of the class distribution per split is provided in Table 1."

as well as

				"The data set served as a reference data set for demonstrating the application of the WBA_n approach in a concrete medical application. It did not implement a dedicated study on its own. The data set was fully anonymized and is accessible under an open access license (CC BY-NC-SA 4.0). Thus, no ethics approval needed to be obtained."
Setting	5	Describe the setting, locations, and relevant dates, including periods of recruitment, exposure, follow-up, and data collection	7	See also comment to "Study design".<br><br>The basic characteristics of the study / the data set are described at the beginning of Subsection "Experimental Setup to Demonstrate the Application of WBA_n". Based on the setup, this does not include detailed information about the study participants, since this was not an own dedicated study.<br><br>In the paper, the following statement is included:<br>"Formally, this represents a retrospective observational / cross-sectional study. No specific hypothesis was addressed regarding the concrete results for the used metrics. Instead, this example served to illustrate the practical applicability of the WBA_n metric and compare it to other weighted evaluation metrics. This demonstrates the steps that have to be performed to set up the cost matrix and apply the WBA_n metric to a concrete use case. This addresses the main objective of the paper, i.e. the definition of the WBA_n metric as a normalized metric that is designed to operationalize the structure required by [10]."
Participants	6	(*a*) *Cohort study*—Give the eligibility criteria, and the sources and methods of selection of participants. Describe methods of follow-up<br>*Case-control study*—Give the eligibility criteria, and the sources and methods of case ascertainment and control selection. Give the rationale for the choice of cases and controls	7	See also comment to "Study design".<br><br>The basic characteristics of the study / the data set are described at the beginning of Subsection "Experimental Setup to Demonstrate the Application of WBA_n". Based on the setup, this does not include detailed information about the study participants, since this was not an own dedicated study.<br><br>Basically, this study represents a cross-sectional study – in this case in an unmatched version. No specific eligibility criteria were applied. Instead, the entire collection was utilized.

		Cross-sectional study—Give the eligibility criteria, and the sources and methods of selection of participants		In the paper, the following statement is included:<br>“No specific eligibility criteria were applied, in this setting.”
		(*b*) *Cohort study*—For matched studies, give matching criteria and number of exposed and unexposed<br>*Case-control study*—For matched studies, give matching criteria and the number of controls per case		N/A – No cohort and no case-control study
Variables	7	Clearly define all outcomes, exposures, predictors, potential confounders, and effect modifiers. Give diagnostic criteria, if applicable	8-10	This study focused on presenting / comparing the outcomes of the metrics in an exemplary medical application. Basically, the following aspects were analyzed and correspondingly described in the methods section:<br>• Comparison of WBA_n to accuracy values as well as other weighted metrics, i.e. AC, weighted kappa score, and CWA.<br>• For WBA_n, analysis of robustness by using a bootstrapping method, including the calculation of the mean and 95% confidence intervals.<br>• Analysis of the dependency of WBA_n on specific parameters in the cost matrix.<br>The diagnostic criteria were discussed in the context of the risk-based approach. This included an analysis of the likelihood and severity of the risks in the concrete real-world application. Additionally, the dependency of the results on the given real-world scenario was analyzed.
Data sources/ measurement	8*	For each variable of interest, give sources of data and details of methods of assessment (measurement). Describe comparability of assessment methods if there is more than one group	7-8	The data was extracted from an existing open access data set. The description of the data sources was included in the paper. The data set only contained the X-ray images of the study participants.<br>Description in the paper:<br>**Dataset Characteristics**<br>Data was recorded across different countries and institutions. It was created from different smaller data sets, namely (i) the Dataset of Tuberculosis Chest X-rays Images [28], (ii) the Labeled Optical

Coherence Tomography (OCT) and Chest X-Ray Images for Classification [27] and (iii) the data set used in [29]. The most important institutions and programs that recorded and collected the images were the National Library of Medicine (U.S.), the Radiological Society of North America, the National Institute of Allergy and Infectious Diseases (Belarus), and (iii) the NIAID TB portal program [33]. No specific eligibility criteria were applied, in this setting.

The sources used to create the dataset differ markedly in origin and acquisition conditions. The class labels are therefore not evenly distributed across these sources. The pneumonia class was derived exclusively from [27], consisting of pediatric radiographs in anterior-posterior (AP) projection from a single center in China, with diagnoses graded by two to three expert physicians. The tuberculosis class was derived from [29] and [28], comprising predominantly adult radiographs in posterior-anterior (PA) projection. The dataset in [29] is an aggregate of the NLM Montgomery and Shenzhen collections, the NIAID TB dataset, the RSNA CXR dataset, and the Belarus dataset (different countries including USA, China, Belarus). The data from [28] was collected at a single hospital in Pakistan. The normal class was drawn from all three sources. Table 2 summarizes the resulting characteristics. No detailed demographic information was available for the data.

The aggregation of different datasets introduces heterogeneity across acquisition site and country, imaging equipment, native resolution and file format, projection, and patient age. For the assessment of the models, it has to be regarded that class membership is confounded with patient population and acquisition protocol: pneumonia cases are exclusively pediatric AP radiographs, whereas tuberculosis cases are predominantly adult PA radiographs. Since pediatric and adult thoracic anatomy differ substantially, a model may in part separate the classes by population- or protocol-specific features rather than by the underlying pathology. Beyond that, the class counts of the dataset available on kaggle exceed the reported

				sizes of the cited sources indicating that data augmentation and/or sampling from the larger underlying data pools was applied during its creation. As the per-image provenance is not documented for the aggregated data set, the images cannot be considered fully statistically independent. This has to be considered when interpreting the bootstrap confidence intervals reported in this paper.
Bias	9	Describe any efforts to address potential sources of bias	8-10	No direct measures could be included to address bias, since we relied on an existing data set without specific information. Regarding the relationship between the data sets and the real-world scenario, the implications were discussed when applying a risk-based approach. In particular, this included an analysis of the expected prevalences in the actual clinical scenario as well as their impact on the calculation of appropriate weights in a risk-based approach.<br><br>In the paper, a description of the data, a description of the real-world scenario, a derivation of the prevalences of the different classes in the real-world scenario and a discussion about these relationships and the associated impact was included.
Study size	10	Explain how the study size was arrived at	7	The overall data set included over 25.000 individual data sets / patients. For development purposes, it was divided into a predefined training, validation, a test dataset. The details are documented in Table 1 of the paper.<br><br>The size was given directly by the available Kaggle data set that was used in our paper. No formal calculation of the study size was used since the study did not address a specific hypothesis to be tested.
Quantitative variables	11	Explain how quantitative variables were handled in the analyses. If applicable, describe which groupings were chosen and why	3 – 10	The study only included an image recognition task that was addressed by different ML models. The parameters used for training the models were provided in Table 2. The methods for calculating the WBA_n as new methods was described in detail. For the other metrics, the relevant literature references were included.

				The data set consisted of three different classes regarding lung diseases, i.e. normal, pneumonia, and tuberculosis. No specific groupings were chosen beyond this.
Statistical methods	12	(*a*) Describe all statistical methods, including those used to control for confounding	8-10	Regarding the evaluation of WBA_n, different statistical methods were applied. This included<br>- K-fold cross validation<br>- Robustness analysis over bootstrap samples<br>- Robustness analysis with regard to variations in the cost matrix<br><br>In the paper, this was documented as follows:<br><br>We applied additional methods to assess the statistical robustness of the results. To demonstrate that the proposed metric is robust to changes in the training dataset, we performed k-fold cross validation where we trained k = 10 models on different folds of the dataset.<br><br>Additionally, we drew B = 1000 bootstrap samples of size N = 2500 with replacement from the test set and calculated the probability that a given architecture is ranked as best model. This served to assess ranking stability across different subsets of the test population. To also examine the robustness towards changing cost structures, we calculated the likelihood that a given model is ranked as best model by the different weighted metrics when scaling the cost matrix with log-normally distributed noise. To create the noise, scaling factors for each entry of the cost matrices C were sampled 1000 times from a log-normal distribution with mean $\mu = 0.0$ and sigma σ of 0.2, 0.5, 1.0, and 1.5.
		(*b*) Describe any methods used to examine subgroups and interactions	7-10	No specific subgroup analysis was performed.<br><br>This was clarified in section “Experimental Setup to Demonstrate the Application of WBA}_n” in the methods section.
		(*c*) Explain how missing data were addressed	7-10x	No specific measures for missing data had to be applied, since this was an already existing data set. Within the particular data sets, there also were no missing data since only the X-ray images were used.

		This was clarified in section “Experimental Setup to Demonstrate the Application of WBA}_n” in the methods section.
(*d*) *Cohort study*—If applicable, explain how loss to follow-up was addressed<br>*Case-control study*—If applicable, explain how matching of cases and controls was addressed<br>*Cross-sectional study*—If applicable, describe analytical methods taking account of sampling strategy	7-10xx	No specific measures for loss to follow-up had to be applied, since this was an already existing data set.<br><br>This was clarified in section “Experimental Setup to Demonstrate the Application of WBAn” in the methods section.
(*e*) Describe any sensitivity analyses	8-10	Regarding the evaluation of WBA_n, different statistical tests were conducted to analyse sensitivity of WBA_n to different phenomenons. This included:<br>- Robustness analysis over bootstrap samples<br>- Robustness analysis with regard to variations in the cost matrix<br><br>Additionally, the dependency of WBA_n on specific parameters of the cost matrix was analyzed.<br><br>In the paper, this was documented as follows:<br><br>“Additionally, we drew B = 1000 bootstrap samples of size N = 2500 with replacement from the test set and calculated the probability that a given architecture is ranked as best model. This served to assess ranking stability across different subsets of the test population. To also examine the robustness towards changing cost structures, we calculated the likelihood that a given model is ranked as best model by the different weighted metrics when scaling the cost matrix with log-normally distributed noise. To create the noise, scaling factors for each entry of the cost matrices C were sampled 1000 times from a log-normal distribution with mean $\mu = 0.0$ and sigma σ of 0.2, 0.5, 1.0, and 1.5.”<br><br>as well as

				“The second scenario was used to demonstrate the dependency of the outcomes regarding particular entries in the modified cost matrix. We systematically varied the relationship between the positive and negative cases or each possible confusion, i.e. each possible misidentification of classes. We systematically applied a factor c in the range 0.1 to 10 to these cases in the reciprocal form ˆc˜i,˜j = c, ˆc˜j,˜i = 1 c for the respectively selected confusions (˜i, ˜j). All other entries in the modified cost matrix were set to ˆci,j = 1 for i ≠ j and $c_{i,j}$ = 0 for i = j.”
**Results**				
Participants	13*	(a) Report numbers of individuals at each stage of study—eg numbers potentially eligible, examined for eligibility, confirmed eligible, included in the study, completing follow-up, and analysed	8-10	The aim was purely creating and evaluating ML models; a fixed dataset was used- It was described in Table 1 as well as in the following description:<br><br>“Data was recorded across different countries and institutions. It was created from different smaller data sets, namely (i) the Dataset of Tuberculosis Chest X-rays Images [28], (ii) the Labeled Optical Coherence Tomography (OCT) and Chest X-Ray Images for Classification [27] and (iii) the data set used in [29]. The most important institutions and programs that recorded and collected the images were the National Library of Medicine (U.S.), the Radiological Society of North America, the National Institute of Allergy and Infectious Diseases (Belarus), and (iii) the NIAID TB portal program [33]. No specific eligibility criteria were applied, in this setting.<br>The sources used to create the dataset differ markedly in origin and acquisition conditions. The class labels are therefore not evenly distributed across these sources. The pneumonia class was derived exclusively from [27], consisting of pediatric radiographs in anterior-posterior (AP) projection from a single center in China, with diagnoses graded by two to three expert physicians. The tuberculosis class was derived from [29] and [28], comprising predominantly adult radiographs in posterior-anterior (PA) projection. The dataset in [29] is an aggregate of the NLM Montgomery and Shenzhen collections, the NIAID TB

				dataset, the RSNA CXR dataset, and the Belarus dataset (different countries including USA, China, Belarus). The data from [28] was collected at a single hospital in Pakistan. The normal class was drawn from all three sources. Table 2 summarizes the resulting characteristics. No detailed demographic information was available for the data. ”
		(b) Give reasons for non-participation at each stage	7-10	The study used an already existing data set as a reference and did not include an own dedicated study. Thus, non-participation was not an issue.<br><br>This was clarified in section “Experimental Setup to Demonstrate the Application of WBA_n” in the methods section.
		(c) Consider use of a flow diagram	7-10	The study used an already existing data set as a reference and did not include an own dedicated study. Thus, there was no need to use a low diagram.<br><br>This was clarified in section “Experimental Setup to Demonstrate the Application of WBA_n” in the methods section.
Descriptive data	14*	(a) Give characteristics of study participants (eg demographic, clinical, social) and information on exposures and potential confounders	7-8	The characteristics of the data set were described in detail in the subsection “Dataset Characteristics” within the methods section. This included the particular components of the data set, their origins, the characteristics of study participants (as far as available). Information about specific exposures and potential confounders was not available in the data sets. Limitation regarding a more detailed description of the study participants were disclosed, accordingly.
		(b) Indicate number of participants with missing data for each variable of interest	7-10	The study used an already existing data set as a reference and did not include an own dedicated study. There were no issues with missing data, since only the available x-ray images were used as a given cohort.<br><br>This was clarified in section “Experimental Setup to Demonstrate the Application of WBA_n” in the methods section.
		(c) *Cohort study*—Summarise follow-up time (eg, average and total amount)	N/A	No cohort study.
Outcome data	15*	*Cohort study*—Report numbers of outcome events or summary measures over time	N/A	No cohort study.

		Case-control study—Report numbers in each exposure category, or summary measures of exposure	N/A	No case-control study
		Cross-sectional study—Report numbers of outcome events or summary measures	7	The study used an already existing data set as a reference and did not include an own dedicated study. No specific outcome events had to be reported. The class distributions regarding the presence of the particular lung diseases are given in Table 1.
Main results	16	(*a*) Give unadjusted estimates and, if applicable, confounder-adjusted estimates and their precision (eg, 95% confidence interval). Make clear which confounders were adjusted for and why they were included	10-12	The unadjusted results are provided in Tables 5 - 7 (regarding metrics), Figure 1 (regarding confusion matrices), and the corresponding text elements in the Results section.
		(*b*) Report category boundaries when continuous variables were categorized	N/A	Regarding the outcomes, no categorization of continuous variables was performed. Regarding the categorization of the likelihood and severity of risks, the quantitative (for likelihood) and semi-quantitative approach (for severity) was used as described in ISO 14971 / ISO/TR24971. The application of this approach was described in the Methods section.
		(*c*) If relevant, consider translating estimates of relative risk into absolute risk for a meaningful time period	N/A	No specific analysis was provided in the results section. However, a systematic translation between the development and the real-world scenario was performed and already described, in the methods section. This was further discussed in the discussion section, based on the results of this study.
Other analyses	17	Report other analyses done—eg analyses of subgroups and interactions, and sensitivity analyses	11-12	The dependency of the WBA_n metric on specific parameters in the cost matrix was analysed as an additional step. This was addressed by test scenario #2.
**Discussion**				
Key results	18	Summarise key results with reference to study objectives	12-15	The basic results are summarized and discussed in the first part of the introduction. Additionally, they are wrapped up in the conclusion section. This includes the following main topics.<br>• Outcome of the practical application of the WBA_n approach<br>• Consistency / variations in the resulting ordering of ML models

				• Comparison to existing weighted metrics<br>• Normalization properties of WBA_n<br>• Dependency of WBA_n on particular parameters in the cost matrix<br>• Relationship between development and real-world scenario<br>• Relationship to relevant regulatory requirements, in particular regarding a consequent implementation of risk management requirements / a risk-based approach
Limitations	19	Discuss limitations of the study, taking into account sources of potential bias or imprecision. Discuss both direction and magnitude of any potential bias	14-15	For the discussion of limitations, a dedicated subsection “Limitations” was integrated. Additionally, an outlook on options for further advances was given in the subsection “Implications for Future Work”. In summary, these topics included the following aspects:<br>• Difficulties regarding the ratings of risks in concrete medical applications<br>• Limitation to one particular medical application as a reference scenario<br>• Missing support of multi-label classification, hierarchical label structures, or sequential decision-making tasks, as well as uncertainty quantification<br>• Missing application of cost-sensitive learning (CSL) approaches in a deeper sense / using a direct approach for CSL, e.g. by integrating weighted metrics / objectives in the training process
Interpretation	20	Give a cautious overall interpretation of results considering objectives, limitations, multiplicity of analyses, results from similar studies, and other relevant evidence	12-15	See the comments in the previous topics (Key Results and Limitations). The outcomes were carefully and cautiously discussed in the sections Discussion and Conclusion. It was checked how far the objectives of the paper / the study were met. The results were compared with other existing approaches using weighted metrics. In particular, this included the fulfilment of basic regulatory requirements for the use of ML models in medical devices. In particular, this addressed the question how consequent a risk-based approach was implemented in these approaches. This included a discussion about the relationship between the development and the real-world scenario regarding the consequent consideration when

				setting up cost matrices. Additionally, extensions towards cost-sensitive learning were discussed. In all these aspects, the impact of the presented approach was carefully addressed and its limitations were discussed.<br><br>Since a new approach was developed and tested using an exemplary reference scenario, the findings could not be compared directly with similar studies or other direct evidence. However, a comparison regarding the application of similar methodologies, i.e. other weighted metrics, was included.
Generalisability	21	Discuss the generalisability (external validity) of the study results	12-15	The general applicability of the provided approach as well as its limitations were discussed. This included a description and discussion how the approach has to be adapted when the underlying medical use case and its circumstances change. In this context, the difficulties for rating the likelihoods and severity of risks were addressed. It was made clear, that this paper only used a single use case to demonstrate the general applicability of the WBA_n approach as a proof-of-concept. The validity regarding concrete applications to other real-world scenarios has to be checked on a case-by-case basis.
**Other information**				
Funding	22	Give the source of funding and the role of the funders for the present study and, if applicable, for the original study on which the present article is based	17/ Cover Letter / Submission Portal	The study had no specific funding. This was declared in the cover letter as well as in the submission portal. The statement was also included in the end statements of the paper.

*Give information separately for cases and controls in case-control studies and, if applicable, for exposed and unexposed groups in cohort and cross-sectional studies.
